# Impact of Antisolvent and Gas Quenching on Wrinkling in Cs_0.15_FA_0.85_Pb(I_0.6_Br_0.4_)_3_ Perovskite Films

**DOI:** 10.1021/acsami.5c07659

**Published:** 2025-10-12

**Authors:** Maria Azhar, Daniele T. Cuzzupè, Yenal Yalcinkaya, Muhammad Irfan Haider, Emilia R. Schütz, Stefan M. Schupp, Yekitwork Abebe Temitmie, Rik Hooijer, Erkan Aydin, Lukas Schmidt-Mende

**Affiliations:** † Department of Physics, 26567 University of Konstanz, Konstanz 78457, Germany; ‡ Department of Physics, Bahir Dar University, Bahir Dar 6000, Ethiopia; ¶ Department of Chemistry, 9183 Ludwig-Maximilians-Universität, Munich 81377, Germany

**Keywords:** tandem solar cells, wide bandgap perovskites, gas quenching, wrinkling, film morphology

## Abstract

The surface topography of the lead halide perovskite layer is a crucial aspect that influences the performance of perovskite solar cells (PSCs). In this work, two different quenching approaches for the crystallization of Cs_0.15_FA_0.85_Pb­(I_0.6_Br_0.4_)_3_ perovskite films are investigated: antisolvent quenching and gas quenching. Both methods, aimed at removing the solvent of the precursor solution and initiating the perovskite nucleation, differ mechanistically and result in different rates of crystallization, which cause surface topographical irregularities, in the form of elongated and elevated structures on the films, termed “wrinkles”. This study shows that antisolvent-quenched perovskite films exhibit a higher density of wrinkles than the gas-quenched counterparts. Pinholes were found along the wrinkles; thus, a higher density of wrinkles leads to more pinholes and to more defective surface topography. The wrinkles also make the surface rougher, hindering homogeneous contact with the adjacent passivation layer and reducing the overall performance of the solar cells. By comparing the two different quenching methods, we obtained insight into the formation of the wrinkles and their effects on the optoelectronic properties of the perovskite films. We identify the gas quenching method as a way to reduce the wrinkle density to achieve better photovoltaic performance in comparison with the antisolvent method.

## Graphical abstract

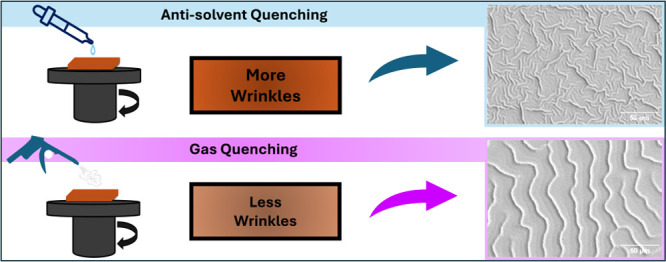

## Introduction

Wide-bandgap (WBG) perovskites (∼1.7–1.9 eV) play a crucial role in all perovskite-based tandem solar cells (TSCs), enabling efficient light harvesting in the high-energy region of the spectrum and unlocking pathways to surpass the single-junction Shockley–Queisser limit. In recent years, various strategies have been explored to improve the performance of WBG perovskite solar cells (PSCs), such as compositional engineering,^1^ surface reconstruction,^2^ additive engineering,^3^ interface engineering,^4^ and defect passivation.^5^ However, one of the most important issues seems to be the optimization of the WBG perovskite films’ morphology. A commonly used approach to improve the morphology of perovskite films, which is suitable for a wide range of perovskite compositions, is the use of methylammonium chloride (MACl) as an additive.^6–9^ However, the addition of the MACl to improve the smoothness and morphology of the films can be problematic in some cases, as methylammonium (MA) can irreversibly react with formamidinium (FA) ions to form n-methyl formamidinium, which may have a detrimental effect on the quality of the perovskite layers.^10^ Therefore, rather than relying on additives to enhance film quality, a more effective and perhaps more insightful approach is to identify the fundamental factors influencing the surface topography and address them directly. One challenge for the uniformity of some perovskite films, depending on the composition, is wrinkling. The wrinkling of perovskite thin films for solar cell applications was first reported by Bush et al. in 2018.^11^ Wrinkling is more common in WBG perovskites and is especially associated with mixed halide perovskites.^12–16^

Few studies have reported that wrinkling improves the photovoltaic performance of devices.^17,18^ Several authors claim that wrinkled structures can increase the light scattering and, thus, improve light absorption and exciton generation. However, excessive wrinkling results in films that are less uniform and rich in localized defects. Rougher films promote, especially when accompanied by pinholes, recombination pathways, reducing the photovoltaic performance.^11^ The stress and strain in the film that cause wrinkling can also affect the stability of the solar cells.^19,20^ Therefore, a moderate amount of wrinkling could be beneficial to the performance of the device and allow for improved light absorption without being overly disruptive. Various approaches to control wrinkles have been explored in the past. For the commonly used antisolvent (AS) quenching method, the chemistry of the AS itself plays a pivotal role in determining the amount of wrinkling in the perovskite film. For example, Kim et al. found that the miscibility of the AS with the host solvent has an impact on wrinkling.^17^ A more miscible AS would lead to faster solvent removal, which causes more wrinkling in the perovskite film. Bush et al. used an interdiffusion method instead of the conventional AS method to control wrinkling. The interdiffusion method involves a two-step spin coating without the use of AS.^11^ Also, the perovskite composition, for example, has an impact on wrinkling.^21^ Generally, simpler compositions, such as pure MAPbI_3_, show less or no wrinkling, whereas wrinkles were observed for various cases in mixed halide perovskites.^17^ Therefore, one way of controlling wrinkling is by changing the composition of the precursor.^22^ Changing the composition, however, alters the optical and electronic properties of the perovskite, especially the bandgap, whereas a well-defined and sufficiently large bandgap is essential for efficient TSCs. In the most common production process, which involves AS quenching, AS is distributed on the spinning substrate following deposition of the precursor solution. As the perovskite precursors are insoluble in AS, crystallization is initiated by its addition. The remaining solvent is then removed during the annealing step. In the gas quenching (GQ) method, on the other hand, no additional solvent is used. Instead, the solvent is removed with a directed stream of inert gas (usually nitrogen) to initiate the crystallization. The process of gas quenching can be divided into three stages: first is gas flow application, second stage is supersaturation and nucleation, and third is crystal growth regulation.^23,24^ These two methods, being inherently different, can determine mechanistic differences and affect the overall performance. The perovskites with the general composition Cs_*x*_FA_1–*x*_Pb­(I_1–*y*_Br_*y*_)_3_ have achieved high PCEs, while showing high stability toward photoinduced phase segregation compared to methylammonium-containing formulations. Therefore, this class of materials is an excellent candidate for WBG perovskite for TSCs applications.^25–27^ The present work was carried out using a perovskite with the composition Cs_0.15_FA_0.85_Pb­(I_0.6_Br_0.4_)_3_, adapted with a slight modification from a method by Li et al.^28^ and further optimized by using ethylenediamine (EDA) as a passivating layer, which has been used in previous reports to improve the overall cell performance.^5^ EDA is only an example of a wide range of molecules commonly used in PSCs to passivate defects in the perovskite layer, for example, used as a surface post-treatment after the perovskite layers have been produced. The reported state-of-the-art efficiency of this perovskite composition is 20.1%.^28^ In this study, we proposed the GQ method as an alternative to the common AS quenching to reduce wrinkling in WBG perovskites. Apart from the reduced wrinkling, the GQ method offers other advantages, such as higher repeatability, reproducibility, compatibility with process upscaling, and cost effectiveness.^29–33^ Huang et al. also found out that an additional gas blowing step to conventional spin coating gives smoother films.^34^ Jiang et al. used a gas quenching method to fabricate stable devices.^35^ Since the solvent removal strategy has an impact on crystallization, the spinning atmosphere and the rate of quenching are crucial for the quality of the perovskite film.^36^ All optimizations for spinning conditions with GQ were part of this study. A pressure of 2 bar (pressure on regulator for nitrogen gun) was chosen as it yielded better results for thin films (absorbance values and luminescence quantum yield values) as well as for PSCs (Figures S1 and S2). We further investigated the onset of crystallization and its effect on surface topographical changes caused by the stress induced by quenching the perovskite on the substrate during deposition. Thereby, the two different quenching methods are evaluated. For GQ, a directed stream of nitrogen gas was used, whereas for the AS method, chlorobenzene was used. The density of wrinkles as a result of these quenching methods, related to the reduction in the degrees of freedom during the perovskite fabrication, is explored. The effect of stress-induced surface topographical changes impacting the grain sizes, crystal orientation, conductivity, and overall performance of devices is investigated. Furthermore, we evaluate thin film properties such as grain sizes, crystal orientation, conductivity, and solar cell performances. Given the significance of the widely used EDA post-treatment for high-performance devices, which we routinely incorporate in our solar cell fabrication protocol, we extended the analysis by employing the EDA passivation and further investigated the interaction of this treatment with wrinkling.

## Results and Discussion

Hereafter, “GQ films/devices/samples” refers to films, devices, or related samples in which perovskite quenching was performed using the GQ method, while “AS films/devices/samples” refers to those quenched using the AS method. The term “perovskite” hereafter refers to the perovskite which is the object of the present study, with composition Cs_0.15_FA_0.85_Pb­(I_0.6_Br_0.4_)_3_ only. The morphology of the perovskite films was examined by scanning electron microscopy (SEM) in top view. As shown in Figure 1a,b, both thin films obtained by AS and GQ exhibit the typical wrinkles observed in those perovskite films. However, the density of wrinkles between the films obtained by the two quenching methods shows notable differences. Remarkably, the thin films prepared by the AS method have a much higher wrinkle density than the films prepared by the GQ method. Multiple samples with the same conditions were characterized to confirm the observation (Figure S3). A similar conclusion can be drawn from the profilometry analysis, as shown in Figure S4.

**Figure 1:**
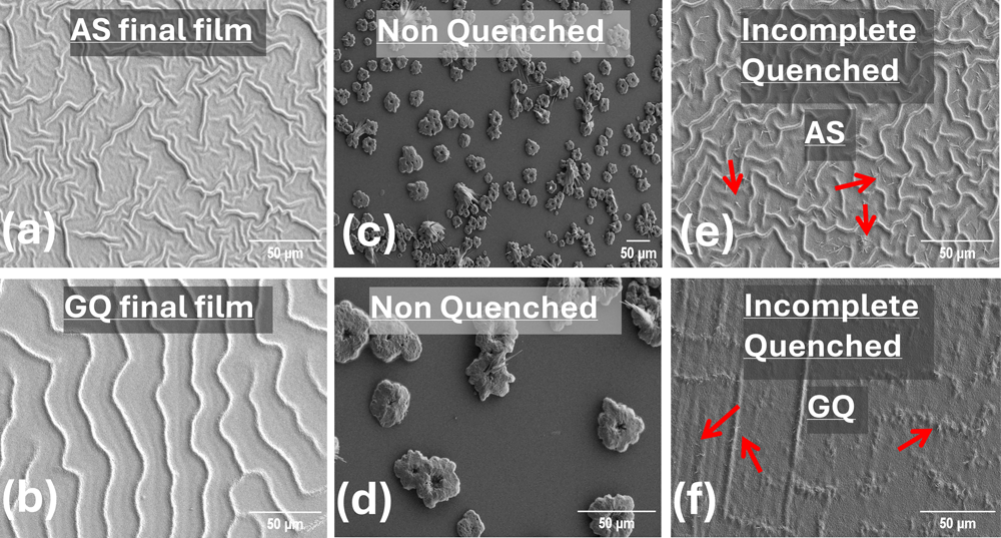
SEM images showing density of wrinkles for (a) AS films, (b) GQ films, (c,d) nonquenched films, (e) incomplete AS quenched films, and (f) incomplete GQ quenched films.

To quantify the approximate density of wrinkles, we performed a systematic analysis of the SEM images of the GQ and AS films shown in Figure 1a,b. A detailed description of the image analysis can be found in the SI. Through binarization and skeletonization of the image, a narrow, one-pixel-broad representation was found for all wrinkles in the analyzed area (Figure S5). Further analysis yielded the cumulative length of all wrinkles in the analyzed area. An approximate total length of 6.5 × 10^4^ μm wrinkles per mm^2^ is found for the AS film, while the GQ film yields roughly 2.5 × 10^4^ μm wrinkles per mm^2^.

This calculation shows that the density of wrinkles in the AS films is almost three times higher than that in the GQ films. In both cases, the presence of pinholes was observed at the edges of the wrinkles, as can be seen in Figure S6a,b, while these are essentially absent in the other regions of the film. Therefore, we infer that the formation of pin holes is correlated with wrinkles. The higher density of wrinkles in the AS films thus results in a higher pinhole density than the GQ films, which is known to contribute significantly to reduced stability, degradation pathways, nonradiative losses, increased ion migration, and enhanced defect density.^37–39^ For optimal photovoltaic performance, the formation of uniform, dense, and pinhole-free perovskite films is essential.^40^ To assess the effect of the GQ and AS quenching on the vertical dimension of the perovskite films, SEM cross-sectional measurements were performed as shown in Figure S7a,b. The process of solvent removal affects the overall morphology of the resulting layer, including its final thickness, when prepared using an identical precursor solution. Contrarily, GQ perovskite films were found to be about 10% thicker than their AS counterparts. This increase in thickness is also reflected in higher absolute values of absorbance intensity (Figure S1a) and photoluminescence (PL) signal counts (Figure S8a) for the GQ films compared to their AS films counterparts.

The prominent difference in the resulting wrinkle densities obtained with identical precursor solutions but two quenching methods serves as a basis to investigate the formation of the wrinkles and their effects in greater detail, which has not yet been fully explored. In particular, a side-by-side comparison of AS and GQ perovskite films is provided throughout this work. First, we discuss the need for a quenching method for the fabrication of a perovskite thin film suitable for solar cells. The morphology of perovskite thin films obtained by spin coating without quenching was observed via SEM, as shown in Figure 1c,d. Isolated and irregularly sparse perovskite crystallites can be seen here. As is apparent from Figure 1, quenching has a controlling effect on the nucleation process. Once nucleation is initiated, perovskite crystallites form and grow into a polycrystalline film. To gain insight into the time scale of wrinkle formation, SEM images of perovskite films at different stages of processing were observed. In Figure 1e,f, the substrates were quenched with either AS or GQ, but the fabrication process was interrupted 5 s after quenching began (i.e., both the rotation and the gas flow were stopped). In the reference process used in solar cell fabrication, the substrate continues to rotate for 15 s after the AS is released; in the GQ process, the gas flow is continuously directed onto the rotating substrates for 15 s. Interrupting both processes prematurely is therefore a way of gaining an insight into the early stages of film formation immediately after the onset of the corresponding quenching method. In the AS case, a large number of wrinkles have already formed, and some partially developed wrinkles are also clearly visible. In contrast, the GQ films do not show fully developed wrinkles in the same period after the start of quenching, but only irregular, vaguely elongated structures. Finally, in Figure S9a,b, the times for quenching and spinning are shown as per the reference procedure, but the films were not annealed. In both cases, fully formed wrinkles were observed, indicating that the wrinkles are formed immediately after quenching and are already fully formed before annealing. In the AS method, the AS is dripped onto the spinning substrates over a period of less than 1 s, while in the GQ method, the forming perovskite films are dried under a directed stream of nitrogen applied continuously for 15 s. The differences in the type and duration of quenching could influence the quenching rate. For this reason, an in situ transmittance setup (experimental details are available in the SI) was used to investigate how the quenching rate affects the crystallization of the perovskite film. In both cases, quenching was performed during spinning, with quenching beginning 20 s after the start of the spinning program, as shown in Figure 2. The decrease in transmittance corresponds to the formation of the dark perovskite film. In both cases, we can first observe the maximum of the transmittance corresponding to the absence of a complete perovskite film, in agreement with the morphology observed for the nonquenched case in Figure 1c,d. During AS processing, a clear decrease in transmittance is observed immediately after the release of the AS, which correlates with the observation of a sudden darkening of the sample that is even visible to the naked eye. This strong change indicates rapid crystallization. In contrast, in the case of GQ, no change in the transmittance is visible in the first 5 s after the onset of the directed gas flow. Thereafter, there is a comparatively gradual decrease in transmittance, indicating a slower crystallization in the GQ films.

**Figure 2:**
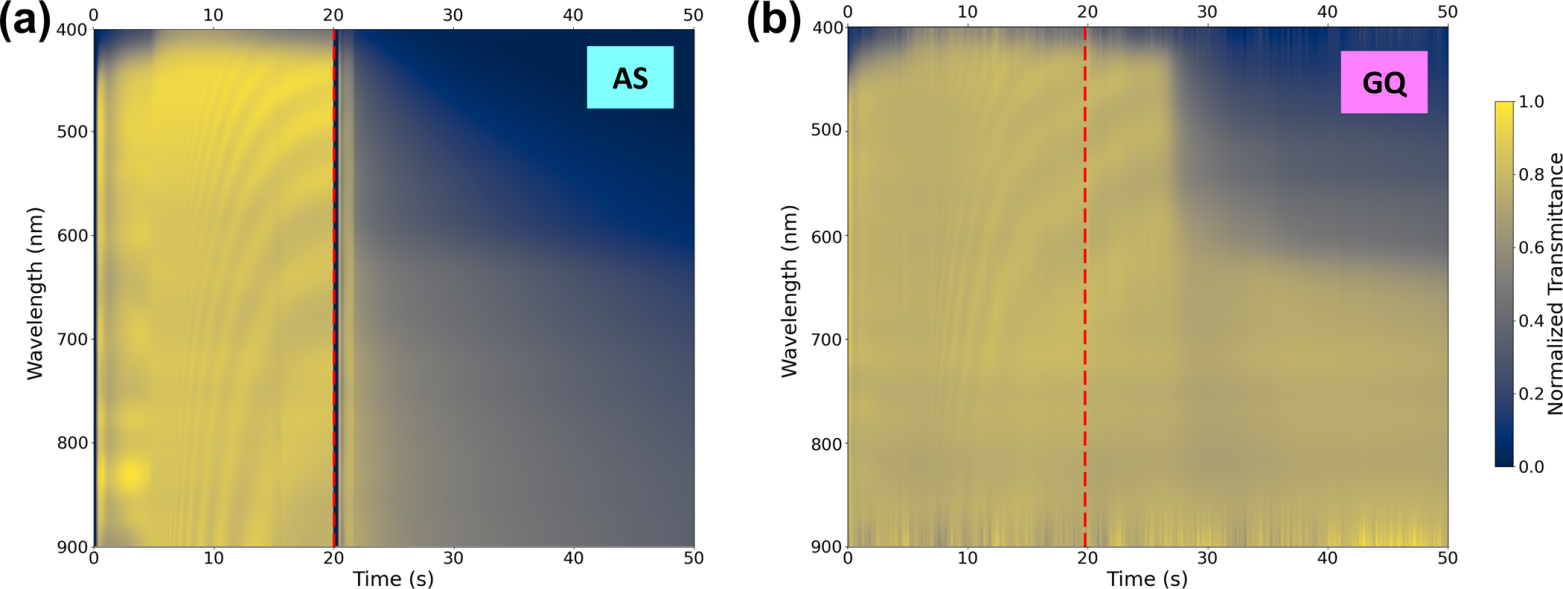
In-situ transmission spectra during the spin coating and quenching process for (a) an AS film and (b) a GQ film. At *t* = 20 s, quenching begins (red dashed line) in both cases.

Furthermore, the in situ transmittance measurements reveal that the film in the AS case has absorption properties at the end of spin time (50 s) and before annealing that are very similar to those of the final (i.e., annealed) stage, with an absorption edge at ∼680 nm (∼710 nm is the absorption edge of the final film (see Figure S1b). Performing the same measurements for the GQ case, the onset of absorbance of the film at the end of the spin time (again before annealing) is found at ∼610 nm. One possible explanation for this is that during AS processing, the Pb-DMSO intermediate complex is rapidly converted to the final perovskite product, because the AS is immediately released and washes away the solvent mixture in the precursor, breaking up this complex, and rapidly extracting DMF/DMSO from the intermediate film.^41,42^ In the case of GQ, however, the solvent is not abruptly rinsed away but slowly dried during the 15 s quench. This results in longer persistence of the Pb-DMSO complex. In other words, the faster the Pb-DMSO complex is cleaved, the faster the crystallization of the perovskite. This faster crystallization could be the reason for the stronger wrinkling observed in the AS film. In an earlier report, Bush et al. suggest that wrinkles form in response to the stress placed on the film during spinning.^11^ The link between wrinkling, stress, and crystallization can be explained by the fact that the mobility of the liquid on the substrate decreases when the AS is dropped onto the spinning perovskite film, causing stress in the film, which then leads to wrinkles. With the GQ method, on the other hand, the movement of the liquid is less restricted due to the slower drying process, resulting in minor stress and consequently fewer wrinkles. Supersaturation in AS forces crystallization to happen quicker, thus leading to an increased number of nuclei and quicker crystal growth.

The composition of the wrinkled regions and the flat regions within the same sample was analyzed by energy-dispersive X-ray spectroscopy (EDX) for samples processed with both quenching methods, as shown in Figures S10 and S11. In both cases, the analysis shows no inhomogeneity in the composition of the wrinkled and flat regions. This corroborates the hypothesis that stressing the films causes localized fluid accumulation leading to wrinkling, but does not lead to differences or gradients in composition. Datta et al. demonstrated that during film formation, the resulting wrinkling arises from a spinodal-like decomposition that generates a pattern of ridges and valleys, where the valleys become predominantly bromide-rich and the ridges iodide-rich.^16^ Our EDX color mapping showed similar results, with iodine being strongly present at the ridges, while bromine was depleted at the ridges but was present in the valleys (Figure S12). To gain insight into the internal morphological features of the wrinkles within the perovskite layer, a focused ion beam (FIB) was used to cut through the section of the AS and GQ perovskite films. An exemplary image of a FIB-cut wrinkle is displayed in Figure S13. No gaps were seen between the perovskite and underlying layer, which suggests that wrinkles are mostly causing surface topographical changes in the perovskite layer. Additionally, it was observed that wrinkles have a trench on their insides. This could be due to the localized reduction in liquid movement that promotes the stacking of two stressed layers that combine to form the wrinkle, while a deeper region remains. Zoomed-out FIB-cut images of both AS and GQ films are shown (Figure S14).

While the stress affects the wrinkle formation, the underlying layer on which the perovskite is deposited can also influence the wrinkle density. To investigate this aspect, we used different charge-selective layers, such as nickel oxide (NiO_*x*_), poly­(3,4-ethylenedioxythiophene) polystyrenesulfonate (PEDOT:PSS), and tin oxide (SnO_2_). Subsequently, the perovskite layers were prepared under identical conditions, and both quenching methods were tested. The SEM images of these layers are shown in (Figure S15). The images show that wrinkle density is affected by the underlying charge transport layers (CTLs); GQ films show less wrinkling than the AS films when deposited onto the same CTLs. The differences observed can be attributed mainly to differences in surface energies due to different underlying layers, which could affect the surface topography of the perovskite film and wrinkle formation. High surface energy HTLs form smoother layers.^43^ Besides, it can be assigned to different coefficients of thermal expansion of the underlying layers.^44^ Different perovskite compositions could present varied degrees of stress based on their lattice constants and their thermal expansion coefficients.

The difference in the rate of crystallization also appears to lead to a slight difference in the grain size between the AS and GQ films. Histograms displaying the grain size distributions obtained by analyzing the SEM images are shown in Figure 3. Both unannealed and annealed films were analyzed. Note that a small grain type (which appears bright in the SEM image) appears on the sample after annealing, as shown in Figure S16. We attribute these brighter grains to crystallized PbI_2_ as previously discussed in the literature.^45^ To confirm this, a perovskite solution was synthesized with a stoichiometric amount of PbI_2_ instead of excess lead iodide (which is used in the reference procedure), and SEM analysis was performed on the film formed after annealing, which showed no bright spots (Figure S17b). Therefore, the bright grains were not included in the grain size analysis as we set our focus on the comparison of the perovskite grains. See grain size analysis in the SI for detailed information. The average perovskite grain size increased after annealing for the GQ and AS samples. However, the GQ samples exhibited slightly larger grains both before and after the annealing process. This could be due to the slower rate of film formation during the GQ process: Since the quenching process is more abrupt overall when using the AS method, more nuclei form and do so in a shorter time. This leads to a denser grain pattern and slightly smaller grain sizes. However, this effect is rather small as the differences between the average values are still within the standard deviations.

**Figure 3:**
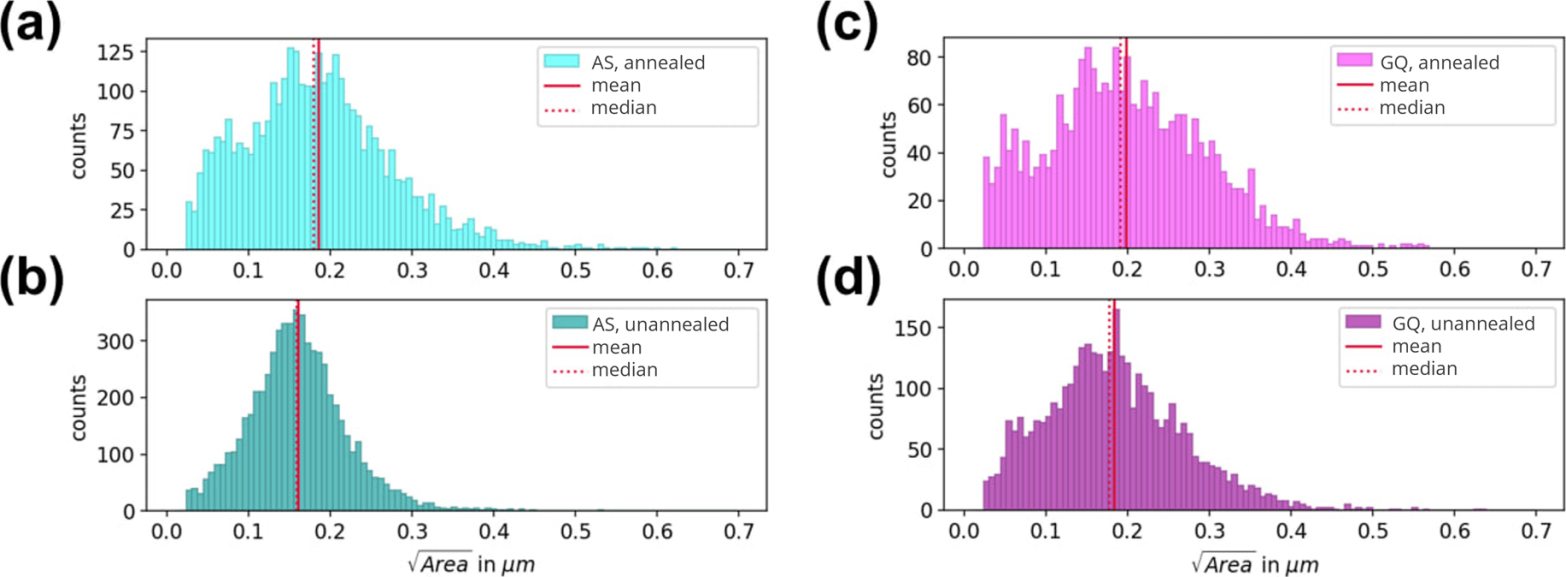
Grain size distribution as extracted from SEM images: (a) AS annealed films, (b) AS unannealed films, (c) GQ annealed films, and (d) GQ unannealed films.

Yang et al. showed that the deposition method of perovskite film has an influence on crystallinity, which was analyzed by grazing-incidence wide-angle X-ray scattering (GIWAXS) measurements of AS and GQ films with and without a passivation layer (EDA). They stated that two-step spin coating is better than single-step spin coating because it can induce controlled nucleation and alter the crystallinity of the perovskite.^46^ To investigate the effects of different quenching techniques with different rates of film formation on the crystalline properties of the perovskite thin films, we performed GIWAXS measurements. To probe the difference between the thin film surface (X-ray probe depth of 3–10 nm) and the bulk (X-ray probe depth of 500 nm), measurements were performed at incident angles of 0.1° and 0.5°, respectively. All samples exhibit the typical narrow PbI_2_ reflection around azimuthal angles χ between ±15° below the first perovskite reflection ring, corresponding to a parallel alignment of this lattice plane with respect to the sample surface (Figure 4). The GQ perovskite films have a relatively higher proportion of PbI_2_ than the AS films, which may be due to different rates of solvent removal in the two quenching methods. Since the GQ process offers gradual crystallization compared to the AS process, PbI_2_ may not fully integrate into the perovskite lattice and instead remain as a separate phase. Most notably, when comparing the AS and GQ samples, a different crystalline orientation can be observed. This is demonstrated in the reduced data by a radial cut integration along the (001) reflection shown in Figure 4c,f, indicated by the white marking in Figure 4b. The difference in intensity between the measurement angles is due to the difference in volume of the analyzed sample. AS samples show preferential (001) orientations peaking at χ = ±62° and ±32° Figure 4c, while the GQ samples peak at χ = ±45° Figure 4f. In addition, the degree of orientation is higher for GQ samples with the EDA surface passivation than without EDA Figure 4f. A preferred orientation along (001) of 45°, i.e., vertically along (111), is associated with higher carrier mobility and stability.^47^ This correlates well with the increase in fill factor (FF) and *J*_SC_ that we observed in solar cells using the GQ perovskite films and passivation treatment for better solar cell performance (see details on device performance below). Moreover, the GQ surface has larger, more distinct crystallites/domains, which can be seen by the pronounced diffraction spots (red spots) shown in Figures S18 and S19, which is known to improve charge carrier dynamics and reduce defect density.^48,49^ These findings agree with the related X-ray diffraction (XRD) measurements performed on thin films (Figure S20), in which the measured diffraction patterns are assigned to a pseudocubic symmetry and the different quenching and annealing conditions show slight differences in the preferential orientation.

**Figure 4:**
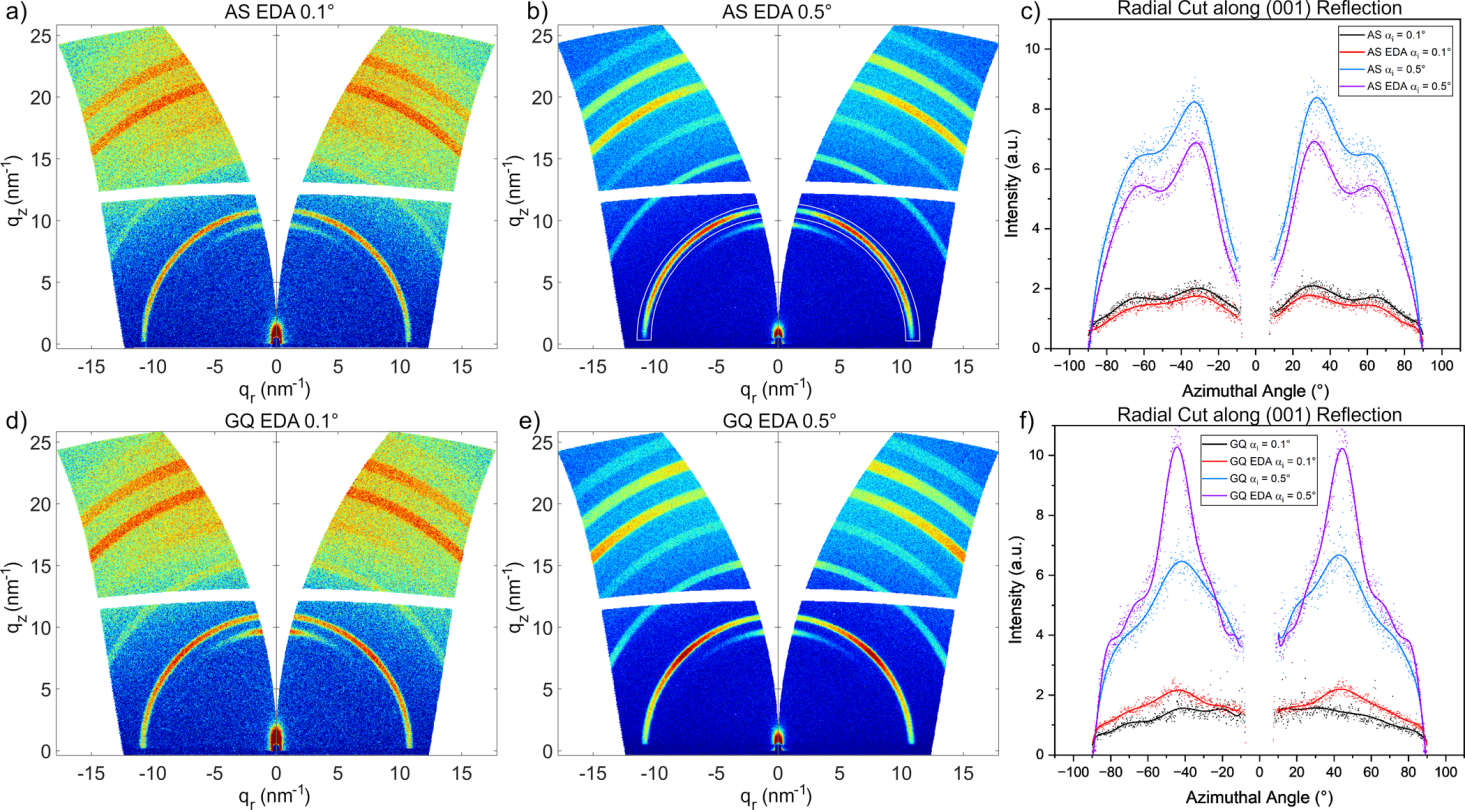
Transformed GIWAXS images for AS (a,b) and GQ, (d,e) samples with EDA surface passivation probing the surface and bulk at incident angles of 0.1° and 0.5°, respectively. Radially cut, integrated data of the (001) reflection for AS passivated and GQ passivated samples (c,f).

The difference in stress on both AS and GQ perovskite films is caused by the rate of solvent evaporation (different for both quenching methods) and mechanical extrusion through the AS drop (which is missing in GQ films). Stress inhomogeneity between the wrinkled and the flat areas could cause additional strain gradients, affecting the film morphology and, in particular, the roughness.^20^ A topographical analysis by atomic force microscopy (AFM) was performed to analyze the surface roughness of both the AS and GQ perovskite films, as shown in Figure 5a,b. The AS film has an RMS roughness of 158.10 nm, while the GQ film has an RMS roughness of 90.17 nm. Different samples were examined to confirm the repeatability of the results, as shown in (Figure S21). This shows that the RMS roughness of the wrinkled regions is higher than that of the flat regions for both AS and GQ films. This leads to generally rougher films for the AS method, where the increased roughness is due to the combined effects of the wrinkle-induced roughness and the higher wrinkle density found in the samples produced using this method, as previously mentioned.

**Figure 5:**
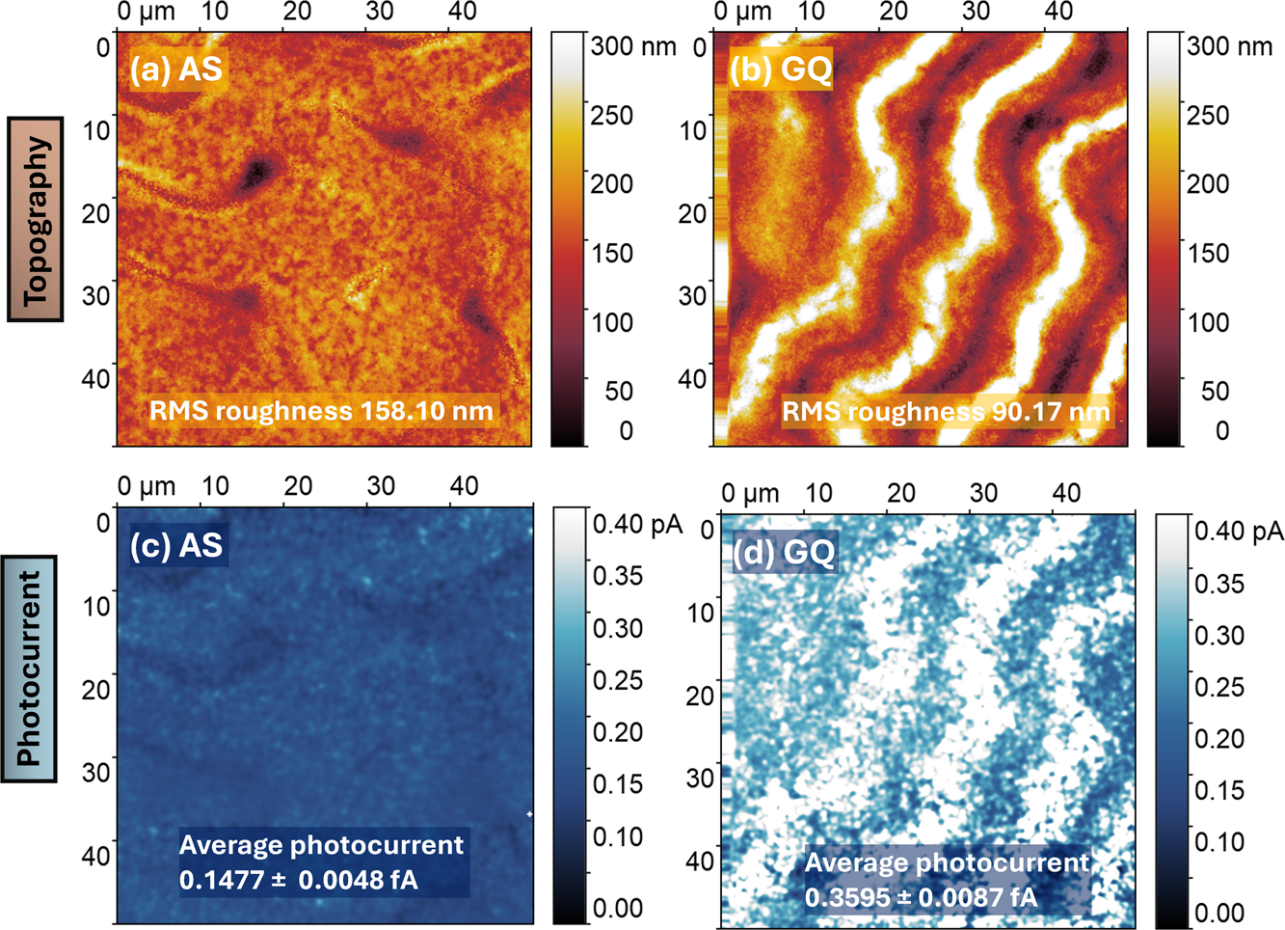
(a) Topography image of AS perovskite film, (b) topography image of GQ perovskite film, (c) current image of AS perovskite film, and (d) current image of GQ perovskite film.

Cheng et al. demonstrated that strain has a crucial impact on the electronic properties of the lead halide perovskite thin films.^50^ For further comparative investigation of the electronic properties of the AS and GQ perovskite films, we measured the local current using conductive atomic force microscopy (c-AFM) in both light and dark mode. The c-AFM maps of the AS and GQ films show differences in the electronic properties. During c-AFM measurements in the dark, we could not detect any current from the AS films. On the contrary, a current is observed for the GQ counterparts, which correlates with higher conductivity values of the latter, as shown in (Figure S22). In the dark mode, no overlay of the topography and c-AFM maps was observed, suggesting that the wrinkles have no effect on the vertical conductivity. Under illumination, the GQ films exhibit a comparatively higher photocurrent and thus higher photoconductivity than the AS ones. The average photocurrent value of the GQ sample is 0.3595 ± 0.0087 fA, and that of the AS perovskite film is 0.1477 ± 0.0048 fA, as shown in Figure 5c,d. There was an overlay between topography and c-AFM maps caused by thickness fluctuations. The higher photoconductivity values exhibited by the GQ perovskite films could be explained on the basis of the lower defect density in these films. Higher luminescence quantum yields were observed for GQ films compared to AS films, both for half-stacks (comprising a perovskite deposited onto an HTL-coated ITO substrate) and for perovskite films deposited directly on microscope glass, which can be related to a comparatively lower defect density for GQ films (Figure S2). The lower number of pinholes for the GQ samples, already discussed in Figure S6, is also related to the lower defect density in these samples. Second, the thicker the films are, the greater the absorption and the higher the photoconductivity values are, and as discussed earlier (Figure S7), the GQ films are comparatively thicker than the AS counterparts when the same conditions are used for the precursor solution and the spin coating process. The overall higher photocurrent in GQ films is consistent with the *J*_SC_ results of the devices described in the next section.

To evaluate the effect of surface passivation in terms of wrinkling behavior and RMS roughness of perovskite films using AS or GQ methods, we fabricated PSCs using both methods. Additionally, each of the two groups of samples was further split into two in order to compare the results obtained with and without ethylenediamine (EDA) passivation for each class of samples. The EDA passivation has proven effective in increasing the device performances across several perovskite compositions.^5,9^ The four solar cell sets (unpassivated AS, unpassivated GQ, passivated AS, and passivated GQ) were analyzed under simulated AM 1.5G conditions. The device structure is illustrated in Figure 6a. Overall, the EDA passivation strategy improved the photovoltaic performance, which confirmed the benefit of this treatment. The PCE of the EDA-passivated GQ devices was ≈2% higher than that of the EDA-passivated AS device. The best efficiency of 18.1% was obtained for the EDA-passivated GQ device. To evaluate the effect of wrinkle density, the influence of the quenching method, the effect of passivation, and the role of the perovskite/C_60_ interface in all scenarios, eight sets of stacks were analyzed with a photoluminescence quantum yield (PLQY) setup. The measurement provided the implied open-circuit voltage (*V*_OC_) corresponding to the quasi-Fermi level splitting (QFLS) as shown in SI (1). The obtained results are plotted in Figure 6b. Looking at the first four plots from the left, it is observed that a *V*_OC_ drop of 40 mV occurs for both the AS and GQ films when a C_60_ thin film is deposited on the perovskite layer, indicating significant nonradiative losses that could be due to high defect density and misalignment of energy levels between perovskite and C_60_.^51–53^ Here, wrinkles do not seem to have a significant effect, as the *V*_OC_ drop is comparable for both quenching methods. With the EDA treatment, effective surface passivation leads to a smaller *V*_OC_ drop for all cases.^5^ If we now compare the *V*_OC_ drop for the EDA-passivated samples with and without the C_60_ interface (four box plots on the right-hand side of Figure 6b), the drop for the AS samples is more pronounced, at around 30 mV, than the same drop for the GQ films, which is merely 10 mV. This could be attributed to the lower wrinkle density shown earlier, which possibly improves the contact between the perovskite and the C_60_ layer. Therefore, both a low wrinkle density and an effective surface passivation are beneficial for device performance, especially in suppressing nonradiative recombination, which could contribute to the overall high *V*_OC_ values found for the EDA-passivated GQ samples. Figure 6c shows the dark *I*–*V* curves of four representative devices. EDA-passivated GQ devices exhibit the lowest leakage current compared to the others. This result is consistent with previous observations, suggesting that the improved film morphology has an impact on minimizing leakage current. Comparable leakage current for both AS and GQ passivated films might suggest that recombination in the film is limited by top surface recombination. Figure 6d shows representative *J*–*V* curves of four device types with forward and reverse scan. Here, all EDA-passivated devices show lower hysteresis than their unpassivated counterparts, which is consistent with previous reports.^5,54,55^ The GQ samples exhibit higher *J*_SC_ values ≈17 mA/cm^2^ (EDA-passivated) and ≈15 mA/cm^2^ (unpassivated) as compared to AS values of ≈16.5 mA/cm^2^ (EDA-passivated) and ≈14.5 mA/cm^2^ (unpassivated), which is consistent with the higher photocurrent values exhibited by the GQ samples in the c-AFM analysis described above. The highest *V*_OC_ value is also achieved by the GQ films (with and without EDA), which agrees well with the fact that the interface between the AS films and C_60_ is more affected by losses than the GQ counterparts, as seen previously. Statistical data of PCE of these four types of devices are shown in Figure S23.

**Figure 6:**
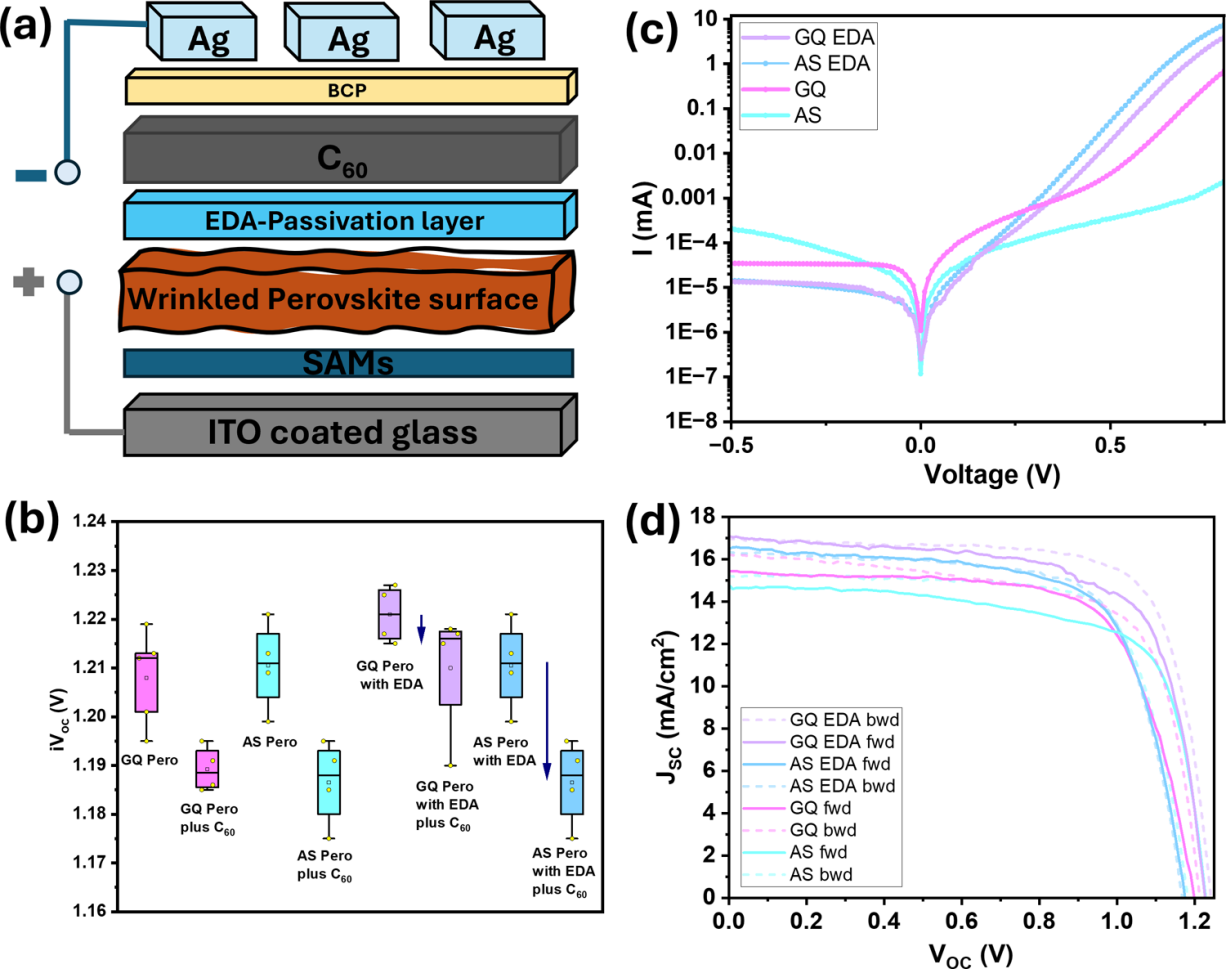
(a) Perovskite solar cell device structure, (b) *V*_OC_ drop from perovskite layer to C_60_ layer, (c) dark *J*–*V* curves of champion devices, (d) *J*–*V* curves of the champion cells recorded in forward and reverse scan directions.

Interestingly, the FF values were also higher in the GQ films. Previous studies have reported that the FF is influenced by the uniformity and morphology of the perovskite film.^56–58^ Figure S24 displays the maximum power point (MPP) tracking, the external quantum efficiency (EQE), and the corresponding *J*_SC,EQE_ values of four representative devices. In general, the GQ samples outperformed the AS samples in our study. It can be concluded that not only the alignment of energy levels, the compatibility with the perovskite, and the electrical conductivity are important, but also the passivation of surface defects works more effectively when the perovskite is smoother in the first place. It is important to note that the repeatability of the photovoltaic parameters of the solar cells is higher for the GQ devices (Figure S25), which is a crucial aspect for the commercialization of PSC technology.

## Conclusions and Outlook

Upon quenching, the formation of wrinkles occurs in Cs_0.15_FA_0.85_Pb­(I_0.6_Br_0.4_)_3_ perovskite films, and these wrinkles are already mature before the annealing step. Antisolvent (AS)-perovskite films exhibit about three times the density of wrinkles as the gas-quenched (GQ) counterparts. Pinholes are predominantly located along the wrinkle boundaries. Consequently, the high wrinkle density in AS films results in a correspondingly high pinhole density in these films. With the same precursor solution and under the same spin coating conditions, the GQ perovskite films were about 10% thicker than their AS counterparts. With in situ analysis, we deduced that the crystallization of perovskite films was faster for the AS method and slower for the GQ method, and that the films obtained via the AS route are closer to their final structure at the end of the spinning process, while the GQ-processed films undergo a slower maturation over time. The reduced crystallization rate of the GQ process also leads to an increased grain size compared with the AS films. Both SEM and GIWAXS analysis revealed larger crystalline domains for the GQ films, and GIWAXS further showed a preferential orientation along the (111) direction, which is beneficial for higher charge carrier mobility and stability. The RMS roughness of the AS films was higher, while the GQ films had higher current values. Without passivation, the *V*_OC_ drop from the perovskite to the C_60_ interface is roughly comparable for either quenching method, but with the passivation treatment, the effect of wrinkles becomes more pronounced. Consequently, we conclude that the passivation treatment is more effective on perovskite films, which are already smoother, improving the interface with the overlying ETL and enhancing overall solar cell performance. The choice of underlying HTLs and the concentration of the precursor solution also influence wrinkling, an aspect not explored in this study but one that could be valuable for future investigation. Efforts to obtain wrinkle-free films may involve exploring different compositions of WBG perovskites in combination with various underlying layers. Excessive roughness can adversely affect the performance of solar cells. Therefore, both a mechanistic understanding of the wrinkling process and the identification of an optimal balance are important for the improvement of solar cell efficiency.

## Materials and Methods

### Materials

For the perovskite solar cell fabrication, ITO-coated glass substrates were acquired from Luminescence Technology Corp (thickness 2 mm, 15 Ω sq^–1^). Formamidinium iodide (FAI) (>99%), formamidinium bromide (FABr) (>99.99%) were purchased from Greatcell Solar. Lead iodide (PbI_2_, 99.99% trace metal basis), MeO-2PACz (>98%) and 2PACz (>98%) were purchased from TCI Chemicals. Cesium iodide (CsI) (99.9%), cesium bromide (CsBr) (99.9%), and lead bromide (PbBr_2_) (99.9%) were purchased from Sigma-Aldrich. Buckminsterfullerene (C_60_, >98.5%) and bathocuproine (BCP) (>99.0%) were acquired from Ossila. Chlorobenzene (anhydrous, 99.8%), isopropyl alcohol (anhydrous, 99.5%), dimethylformamide (99.8%, extra dry), and dimethyl sulfoxide (99.7%, extra dry) were purchased from Sigma-Aldrich. Absolute ethanol was purchased from VWR. All chemicals were used without further purification or processing.

### Fabrication of Perovskite Solar Cell

The WBG PSCs were fabricated on clean ITO-coated glass substrates. The substrates were cleaned by ultrasonication in soapy water, acetone, and isopropyl alcohol for 15 min each, and then they were carefully dried and placed in a UV/Ozone cleaner (Ossila) for 15 min. A 0.5 mM equimolar (50:50) mixture of self-assembled monolayers (SAMs) MeO-2PACz and 2PACz in ethanol was spin-coated onto the ITO substrates in a nitrogen-filled glovebox (3000 rpm for 30 s), followed by annealing at 100 °C for 10 min. The WBG perovskite solution (1.2 M) was prepared by mixing the following precursors at room temperature in a glovebox: CsI (28.1 mg), CsBr (15.3 mg), FAI (105.2 mg), FABr (51 mg), PbI_2_ (358.5 mg) (8% in excess) and PbBr_2_ (176.2 mg) in 1 mL of DMF:DMSO 4:1 in volume. The solution was completely dissolved and used without any filtering. The perovskite solution (55 μL) was deposited onto a static ITO substrate and spin-coated for 5 s at 1000 rpm, followed by 45 s at 3000 rpm. N_2_ flushing started after 20 s of spinning of the precursor solution, and the substrates stayed under N_2_ flushing for 25 s (ending when 5 s of total spinning time were left). For the gas stream, parameters such as the distance between the gas nozzle and the substrate, the angle of the gas distribution, the quenching time, and the nozzle size were carefully considered in this study. The gas pressure was tuned between 2 and 5 bar (reading at the regulator of the nitrogen source). The optimal pressure (2 bar) was used to produce thin films for PSC devices and separately for thin film characterization. For surface passivation, EDA (0.75 mM) in IPA was spin-coated dynamically at 5000 rpm for 20 s (70 °C annealing for 10 min). Stepwise quenching conditions during spinning of the perovskite film for the AS and the GQ routes are shown in a schematic representation. The first step described in (a) and (d) is similar for both; it is the deposition of 60 μL of perovskite solution onto the already SAM-coated film. The basic difference lies in the next step, as shown in (b) and (e). In (b), the antisolvent is distributed during spinning after the 20 s of spinning time has passed, whereas in (e), the nitrogen gas is blown by the gun after 15 s of spinning time has elapsed, and there is a constant flow of nitrogen gas directed to the films for about 15 s. After the quenching step, the films were dried at 100 °C for 10 min (Figure S26) for both methods. The substrates were then transferred into a thermal evaporator (Oerlikon), where 25 nm of C_60_ were deposited at a rate of 0.1 Å/s, followed by 7 nm of bathocuproine (0.1 Å/s) and finally 100 nm of silver (1 Å/s). All thermal evaporation procedures were carried out under a vacuum <8 × 10^–6^ mbar.

### Characterization

Scanning electron microscope (SEM) images were acquired with a Gemini 500 FESEM system from Zeiss equipped with an in-lens detector. For grain size analysis, we have performed SEM measurement using an acceleration voltage of 5 kV. Under these conditions, the resolution is 1–1.5 nm for the InLens detector and 2–3 nm with the SE2 detector. Since the width of the distribution of grain sizes is much larger than the resolution of the SEM images, we do not expect the grain size analysis to be limited by the SEM resolution but rather by its own width and the analysis method. We have used two magnifications (15kX and 20kX) for grain size analysis. An energy-dispersive X-ray (EDX) Ultim Max 100 setup for analysis of emitted X-rays, capable of detecting light elements starting from beryllium, was used for EDX analysis. Optical profilometry was performed using a DektakXT Advanced System surface profiler, manufactured by Bruker, that can accommodate large samples with a maximum thickness of 40 mm. The motorized stage can translate in the *X*–*Y* direction for 150 × 150 mm with a maximum scan length of 55 mm and a vertical range of 1 nm–1000 μm. Steady-state photoluminescence spectroscopy (SSPL) was performed with a FluoTime300 setup from PicoQuant, using a 405 nm excitation laser. The repetition rate was set at 40 MHz. The external quantum efficiency (EQE) was measured using an Enlitech QE-R setup with a 75 W Xe lamp as the light source, operated in AC mode with a chopper frequency of 16 Hz and without light bias. A NIST-traceable, certified Si detector was used to calibrate the monochromatic light intensity. UV–visible-NIR absorption spectroscopy was used to measure the spectra of the perovskite layers. The measurements were carried out with a Cary 5000 UV–vis-NIR spectrophotometer from Agilent Technologies.

The focused ion beam (FIB) milling was performed in a Zeiss CrossBeam 1540XB FESEM. The gallium ion current was set to 50 pA at a chamber pressure of about 3 × 10^–6^ mbar, and the energy of the ions was 30 keV. The milling power was fixed at a value that corresponds to a milling depth of 1.5 μm in pure silicon. Photoelectron spectroscopy in air (PESA) was carried out using an AC-2 system purchased by Riken Instruments. The quantity of light correction was performed before starting the measurement.

The atomic force microscopy (AFM) and conductive atomic force microscopy (c-AFM) measurements were carried out on a Park NX-10 atomic force microscope. AFM measurements were performed with an OTESPA-R3 cantilever (Bruker) with a spring constant of 26 N/m and a free resonance frequency of 300 kHz. c-AFM measurements were carried out with a SCM-PIT-V2 cantilever (Bruker) with a spring constant of 3 N/m and a free resonance frequency of 75 kHz. During c-AFM measurements, a bias of 2 V was applied to each sample. For photo-c-AFM measurements, an additional laser at 785 nm with 5W power was used to illuminate the samples.

X-ray diffraction (XRD) measurements were performed using a Bruker D8 Discover diffractometer (Cu K α_1_ radiation, λ = 1.5406 Å) equipped with a LynxEye detector and a collimator of 2.0 mm.

The photoluminescence quantum yield (PLQY) was measured using a LuQY Pro setup from Quantum Yield Berlin equipped with a 550 nm long-pass filter between the sample and the detector. GIWAXS measurements were carried out on an Anton-Paar SAXSpoint 2.0 with a Primux 100 microfocus source with Cu–Kα1 radiation (λ = 1.5406 Å) and a Dectris Eiger R 1 M 2D Detector. The sample–detector distance was 121 mm, and the incident angles were 0.1° or 0.5°, as indicated for each measurement. Corrections and data analysis were performed with SAXSanalysis. Current density–voltage (*J*–*V*) characteristics curves of the PSCs were recorded under 1 sun illumination with a step size of 0.01 V and a delay of 0.01 s in a nitrogen-filled glovebox with an assembly consisting of a Keithley 2400 SMU, LOT 300 W xenon solar simulator, and a SI-4 calibrated with a Fraunhofer ISE-certified Si reference diode equipped with a KG5 filter window. The maximum power point (MPP) tracking algorithm, developed by Zimmerman et al.,^59^ was employed for a reliable PSC measurement. Light intensity-dependent *J*_SC_ and *V*_OC_ measurements were performed by using the same setup and a modified mask containing various density filters. Transmission measurements during spin coating were conducted using a lab-built setup, as illustrated schematically in Figure S27.

## Supplementary Material



## Notes

Details on in situ transmission setup and grain size analysis, optimizations to the gas quenching technique, and all film characterizations in detail (PDF)
