# Supplementary material for: Impact of Antisolvent and Gas Quenching on Wrinkling in Cs0.15FA0.85Pb(I0.6Br0.4)3 Perovskite Films
Source: ACS Appl Mater Interfaces. 2025 Oct 12;17(42):58501–11. doi: 10.1021/acsami.5c07659 (PMC12557210; doi:10.1021/acsami.5c07659)
Supplement: Supplementary file 1 [file am5c07659_si_001.pdf]

Supporting Information: Impact of anti-solvent and gas quenching on wrinkling in $Cs_{0.15}FA_{0.85}Pb(I_{0.6}Br_{0.4})_3$ perovskite films

Maria Azhar,† Daniele T. Cuzzupè,† Yenal Yalcinkaya,† Muhammad Irfan Haider,† Emilia R. Schütz,† Stefan M. Schupp,† Yekitwork Abebe Temitmie,†,‡ Rik Hooijer,¶ Erkan Aydin,¶ and Lukas Schmidt-Mende*,†

†*Department of Physics, University of Konstanz, Konstanz, 78457, Germany*

‡*Department of Physics, Bahir Dar University, Bahir Dar, 6000, Ethiopia*

¶*Department of Chemistry, Ludwig-Maximilians-Universität, Munich, 81377, Germany*

E-mail: lukas.schmidt-mende@uni-konstanz.de

Gas quenching parameters

A schematic illustrating the different steps of processing the perovskite films with the two quenching method is described here (Figure S26). Pressure used during gas quenching plays a very important role. A nitrogen gun with circular nozzle with diameter d = 1 mm (regulator pressure around 2.5 bar) was held 5 cm above the spinning substrate, perpendicularly. Film characterizations include UV-Vis-NIR spectra, showing the highest absorption values for GQ films at 2 bar. The highest LuQY values were also reached by GQ films at 2 bar. Full devices at various pressures were made to compare the efficiency of devices processed with various quenching pressures. The PCE at 2 bar was higher. Lower pressure than 2 bar resulted

largely in non-uniform films. Therefore, a range of 2 bar to 5 bar was explored. Some cracks were also observed occasionally with 5 bar pressure.

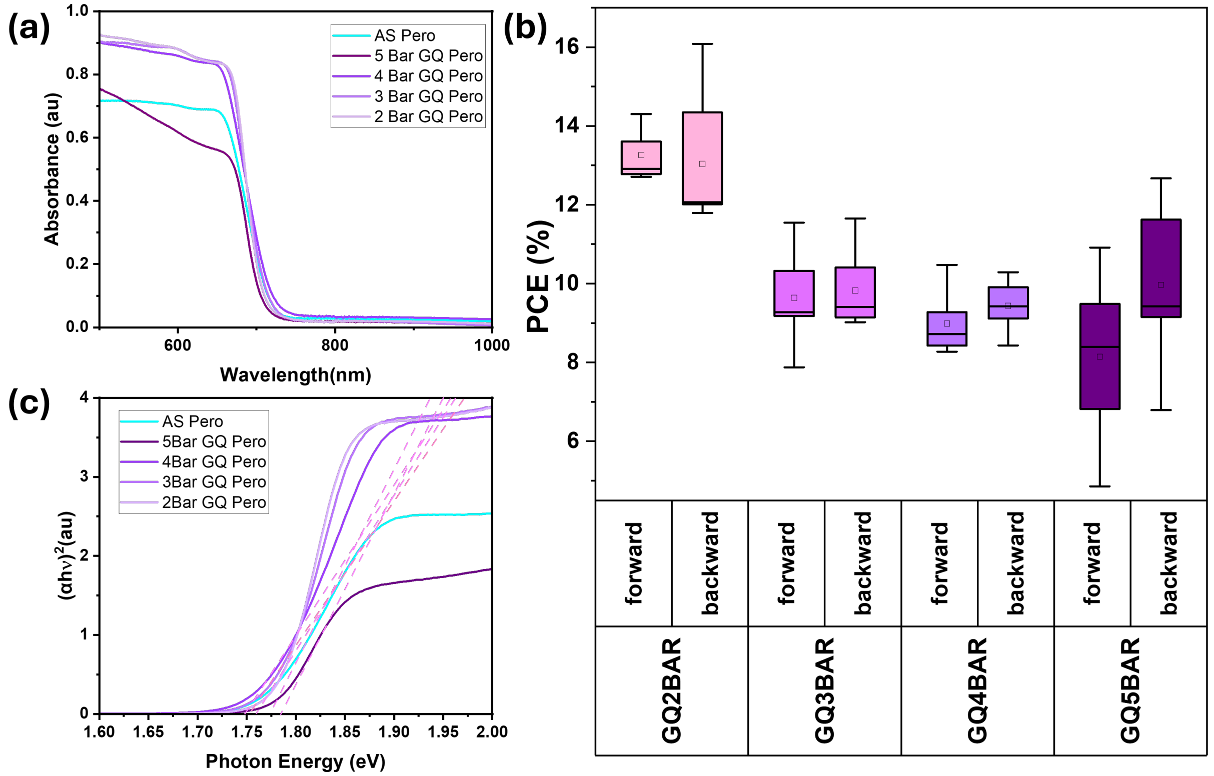

Figure S1: (a) Absorbance data of AS and GQ films at various pressures for half stacks (ITO/HTL/perovskite). (b) PCE data of AS film and GQ film at various pressures. (c) Band edges data of AS film and GQ film at various pressures for half stacks (ITO/HTL/perovskite) with error value (Δ_{max} = 0.025 eV).

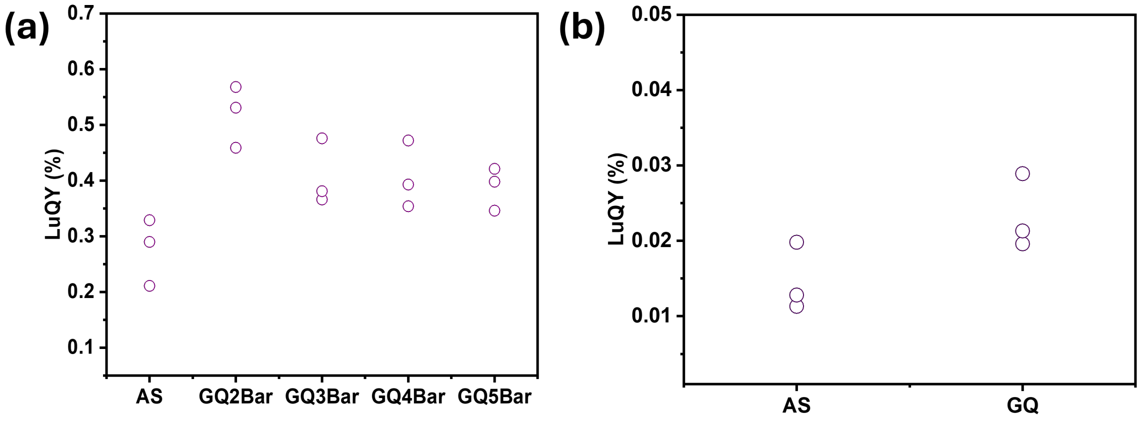

Figure S2: (a)LuQY values of AS films and GQ films at various pressures for half stacks (ITO/HTL/perovskite). (b) LuQY values of AS films and GQ films at various pressures for perovskite only on glass.

$$\mathrm{QFLS} = k_B T \cdot \ln\left(\frac{J_{\mathrm{rad}}}{J_{o,\mathrm{rad}}}\right) = k_B T \cdot \ln\left(\mathrm{PLQY}\frac{J_G}{J_{o,\mathrm{rad}}}\right) \quad (1)$$

$$= \mathrm{QFLS}_{\mathrm{rad}} + k_B T \cdot \ln(\mathrm{PLQY}) \quad (2)$$

where $k_B T$ is the thermal energy, J_{rad} is the radiative recombination current density and J_{G} is generation current.

Density profile of wrinkles

Different AS and GQ films were analysed to confirm the authenticity of results. AS films always show higher density of wrinkles than GQ films.

Optical profilometry was used to investigate the density of wrinkles in AS and GQ-films. The number of peaks depicts the comparatively heightened area which is correlated to wrinkles here (Figure S4).

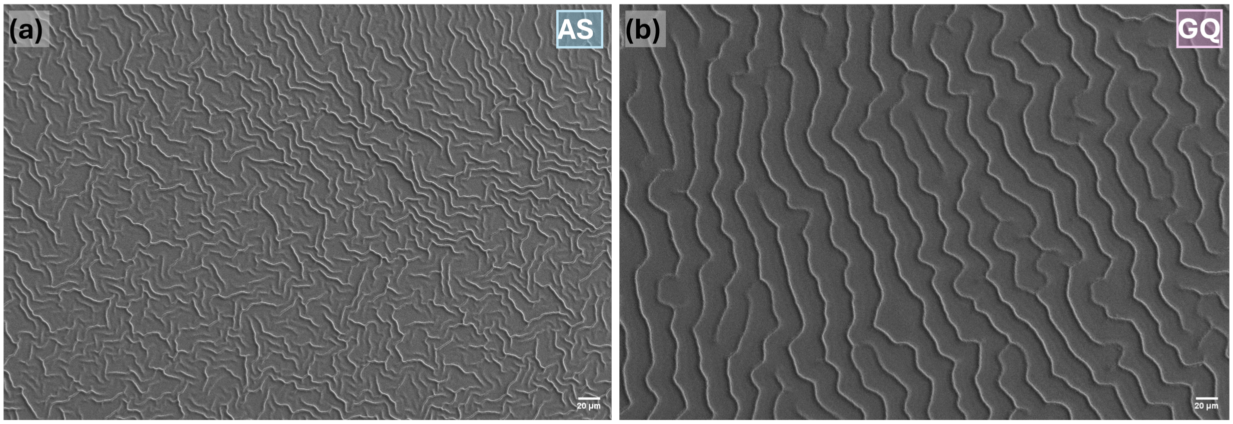

Figure S3: (a) AS perovskite film. (b) GQ perovskite film.

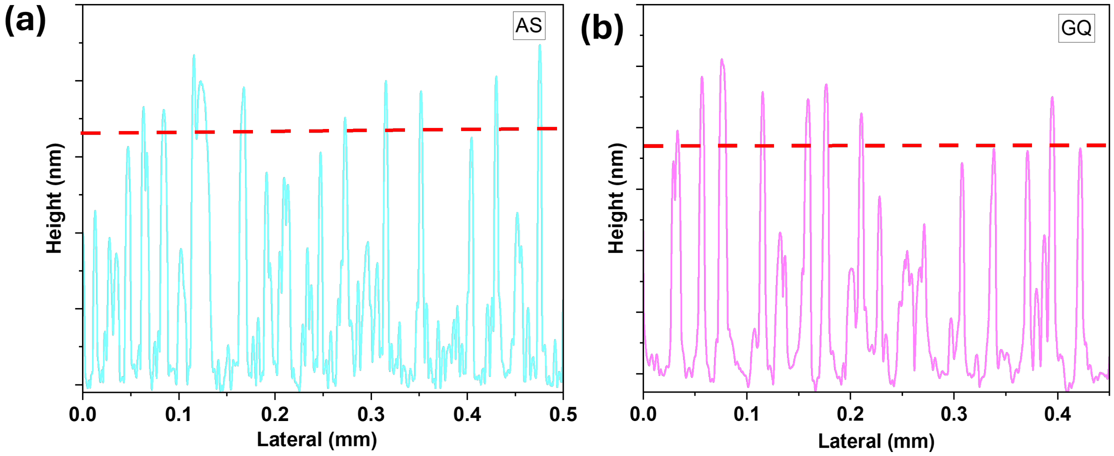

Figure S4: (a) Profilometer analysis of AS film. (b) Profilometer analysis of GQ film.

Quantitative analysis of wrinkling

To analyze the amount of wrinkling on the GQ and AS films, an image analysis of SEM images was performed. The wrinkles are clearly distinguishable in the SE2 images as comparatively lighter areas (see Figure S5 (a) and (d)). We exploited this fact and thresholded the SEMs, obtaining binary images with true values everywhere the pixel brightness I is above a threshhold $t < I$ and false values everywhere $t > I$. The threshold was calculated using Otsu's thresholding method. Otsu's method is a widely-used, automatic threshold

selection technique that maximizes the inter-class variance between the two classes of pixels, determining an optimal threshold value from the image histogram.[1] To optimize the resulting images and represent wrinkling as well as possible, the images were first opened (to remove bright spots unconnected to any larger wrinkle), then closed (to close gaps within one wrinkle that might occur during opening). The resulting binary images (Figure S5 (b) and (e)) reflect the wrinkles reasonably well, while still containing some artifacts, especially for the AS film. Finally, these binary images are skeletonized, reducing the structural representation of all binary objects to one-pixel-wide skeletal forms. Skeletonization is a morphological operation that preserves the topology and connectivity of the original binary image while stripping away redundant pixel information. We utilized the Lee's algorithm for skeletonization.[2] To fully remove all smaller artifact areas, skeleton segments shorter than 50 pixels were dismissed. The final representations of the wrinkles are shown in Figure S5 (c) and (f). Each wrinkle is represented by a line of single pixels. Counting the number of pixels on the wrinkle skeletons now allows us a quantitative analysis of the wrinkling in both films. Roughly 3800 pixels for GQ, and 10100 pixels for AS are situated at the top of the wrinkles. That translates to a cumulative wrinkle length per area of $0.025\,\mu m\,\mu m^{-2}$ for the GQ sample and $0.065\,\mu m\,\mu m^{-2}$ for the AS sample: There is almost three times more wrinkling in the AS film than in the GQ film.

Note that, while there are still some artifacts to this analysis, our qualitative observations regarding the higher incidence of wrinkling in the AS films are quite robust. The amount of wrinkling in the GQ samples is likely to be overestimated (there are several side branches to the wrinkles visible in Figure S5 (f) that are not truly new wrinkles, but artifacts stemming from the processing methods), while it is likely underestimated in the AS films (only the most prominent wrinkles could be resolved clearly, despite there being less pronounced wrinkles visible in Figure S5 (a) in between the larger ones).

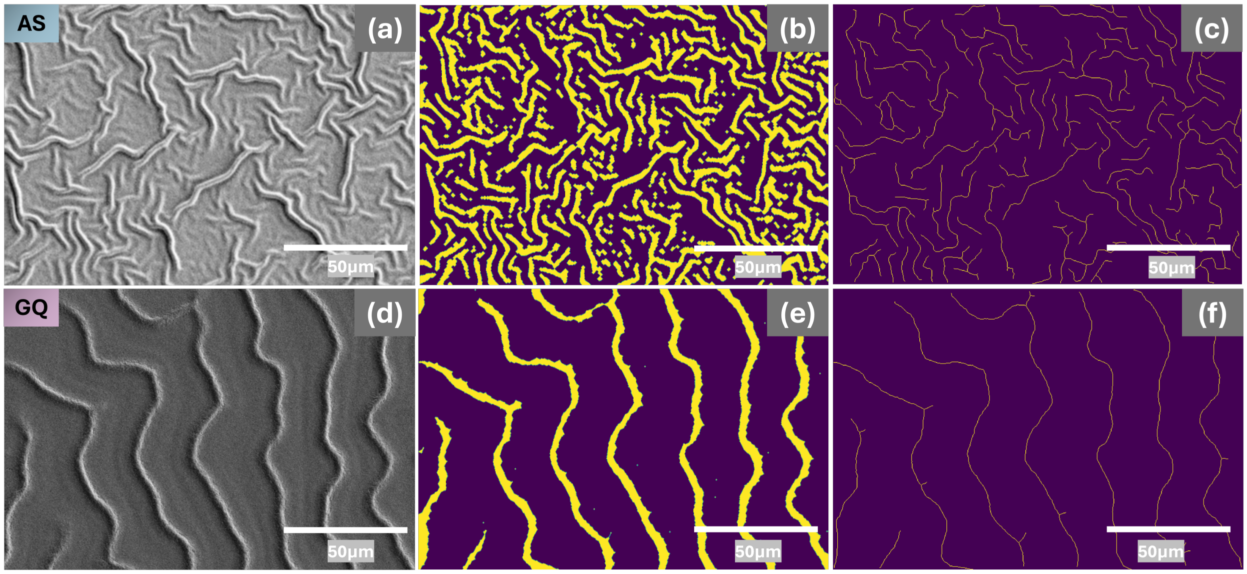

Figure S5: Quantitative analysis of wrinkling on SEM images. SEM images (a,d) are thresholded to yield binary images (b,e). Because of the relative higher brightness on top of the wrinkles in the SEM, wrinkles are well-visible on these binary images. Skeletons of these binary structures (c,f) trace the observed wrinkles reasonably well. The total amount of wrinkling per area can be calculated from the number of pixels representing wrinkles in the skeletonized images.

Film characterization

Steady-state PL measurements were conducted at different spots for AS film and GQ film. Some areas gives lower PL intensity than other areas at similar film because PL intensity is different at wrinkled and flat surface. Overall, the GQ films shows comparatively higher PL intensity counts than the AS films (Figure S8).

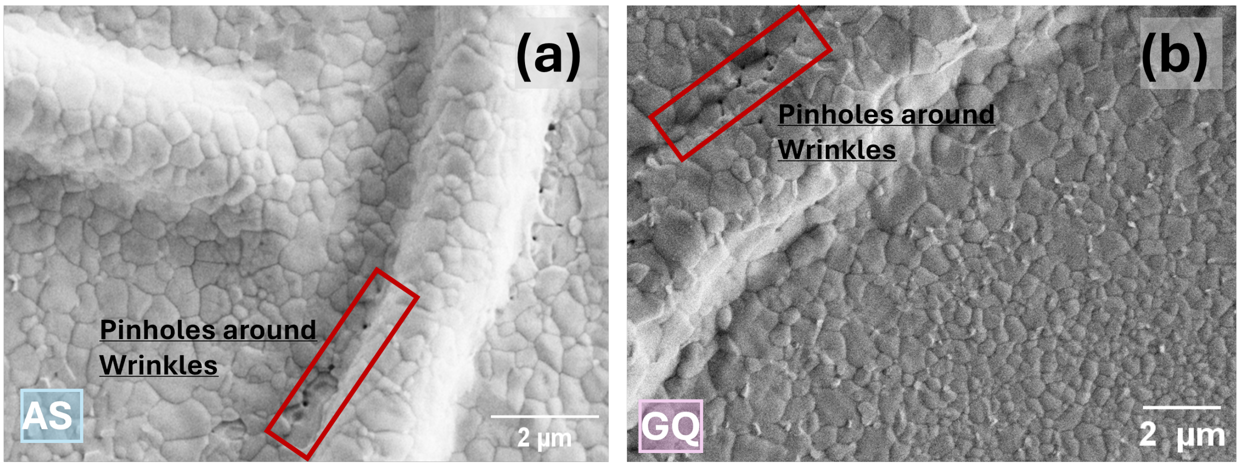

Figure S6: SEM images showing (a) pinholes around AS and (b) GQ perovskite films.

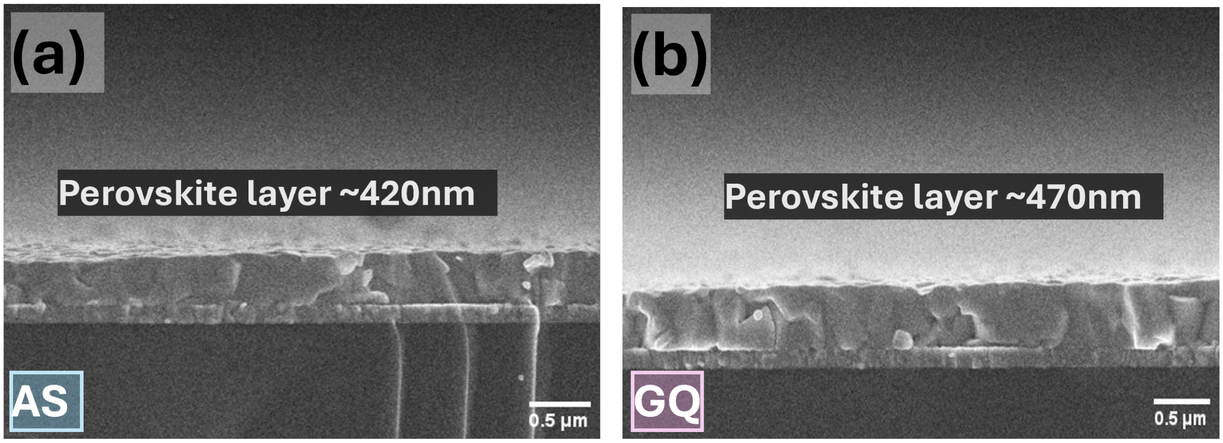

Figure S7: Cross-section SEM showing thickness of (a) AS and (b) GQ perovskite layer.

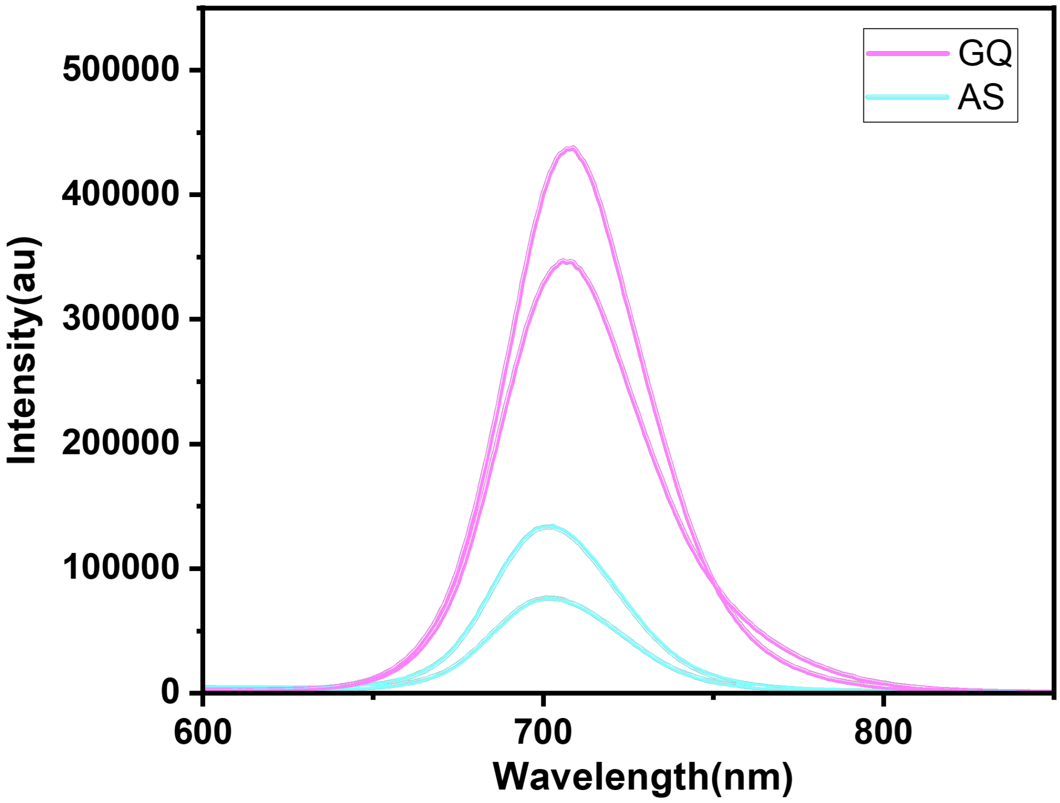

Figure S8: Steady-state photoluminescence emission spectra of AS and GQ film for half stacks (ITO/HTL/perovskite) at different spots.

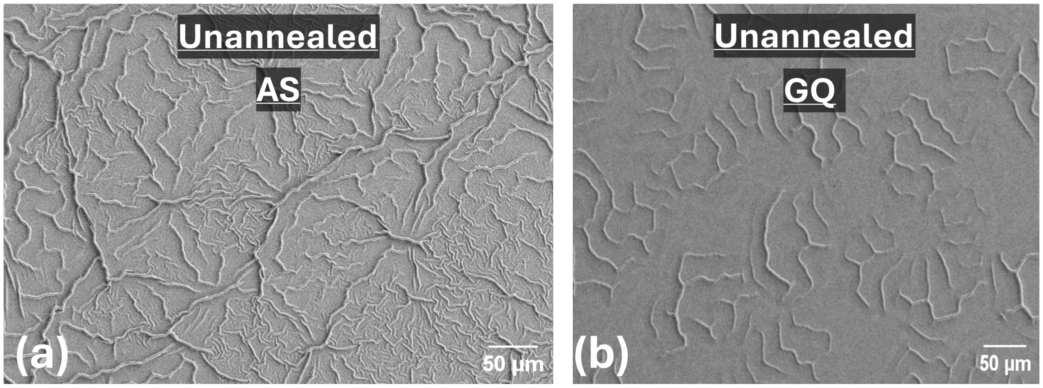

Figure S9: SEM images of perovskite films: (a) unannealed AS and (b) unannealed GQ .

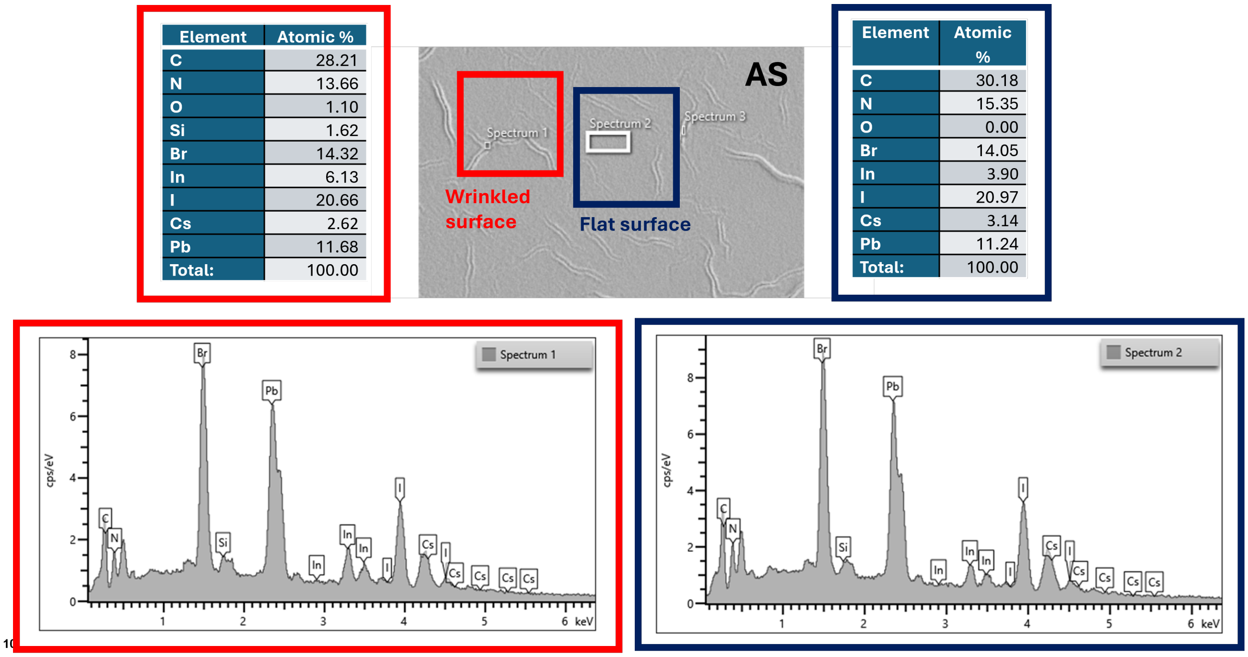

Figure S10: EDX analysis of wrinkled and flat surface on AS film for half stacks (ITO/HTL/perovskite).

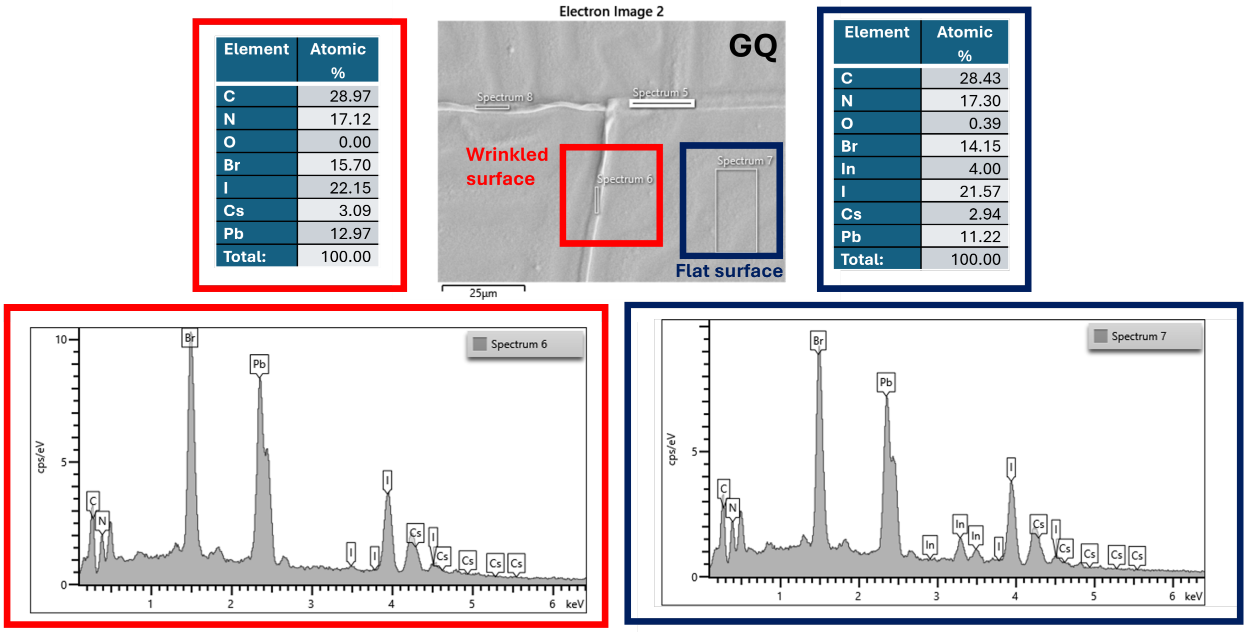

Figure S11: EDX analysis of wrinkled and flat surfaces on GQ film for half stacks (ITO/HTL/perovskite).

The purple square in Figure S12 highlights regions with both wrinkled and flat surfaces, along with their corresponding elemental distributions. Note that the wrinkled and flat surfaces represent two distinct features of the film, whereas the ridges and valleys (described in the main text) are characteristics within the wrinkled region.

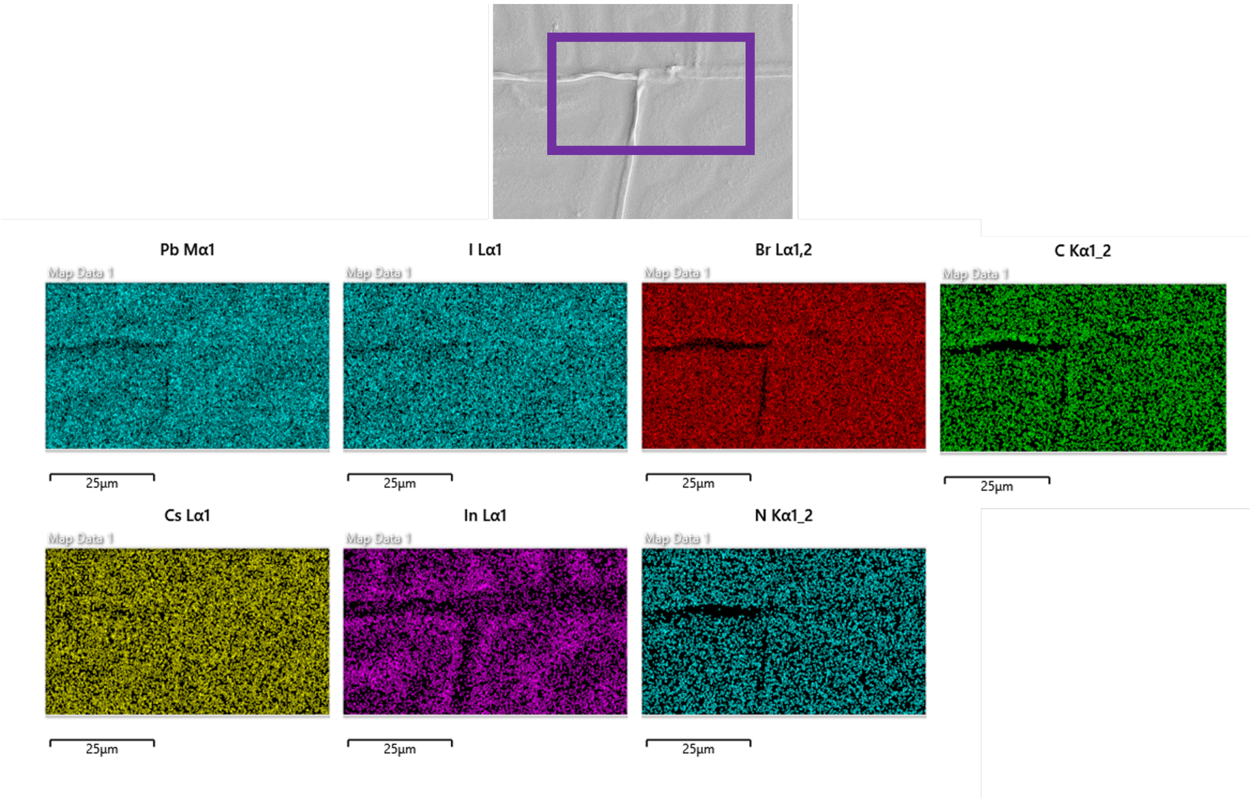

Figure S12: EDX color mapping showing the spatial distribution of relevant elements in a portion of a perovskite film.

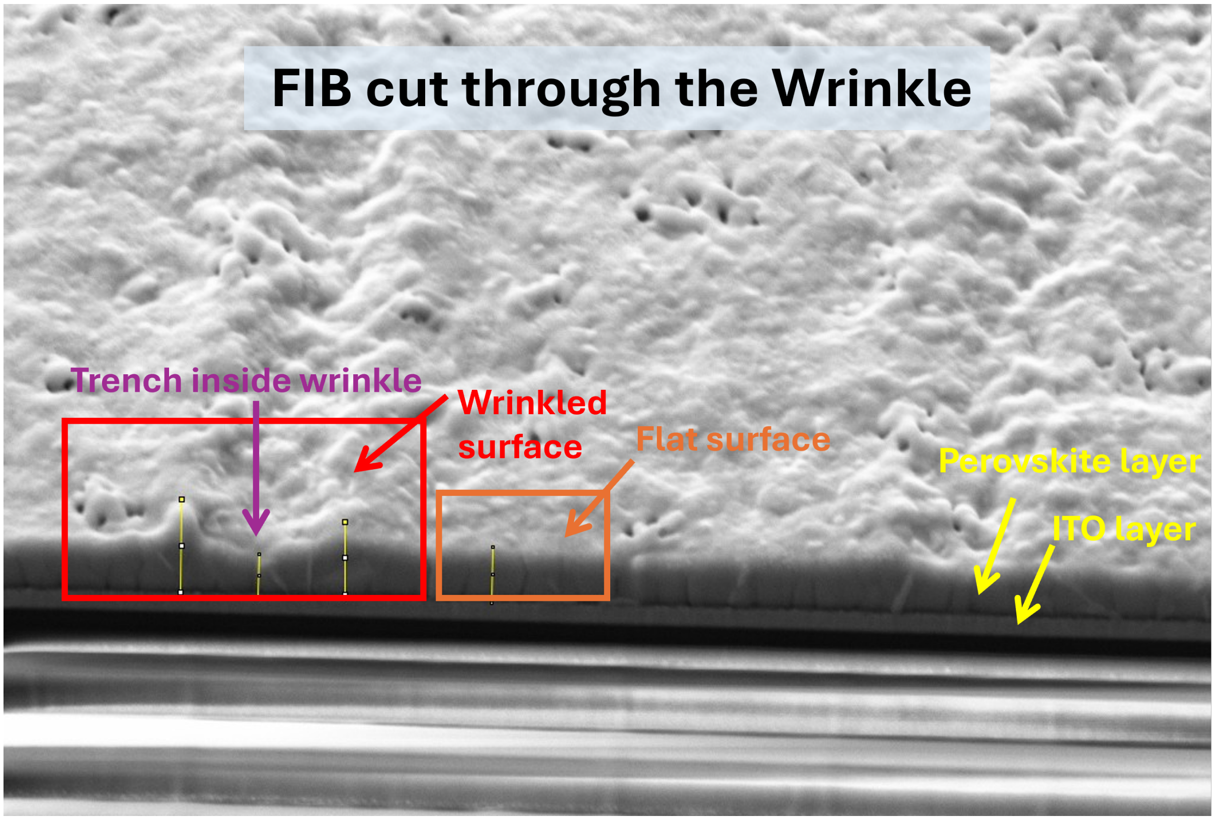

Figure S13: FIB-cut cross section of an AS film showing trench inside wrinkle for half stack (ITO/HTL/perovskite).

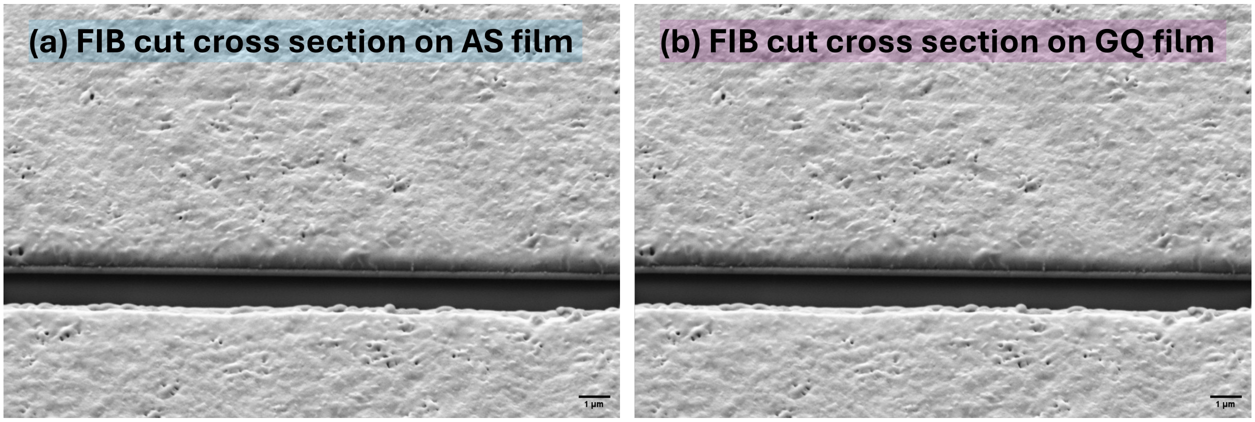

Figure S14: (a) FIB-cut cross section of a AS film deposited on a ITO/HTL stack, (b) FIB-cut cross section of a GQ film deposited on a ITO/HTL stack.

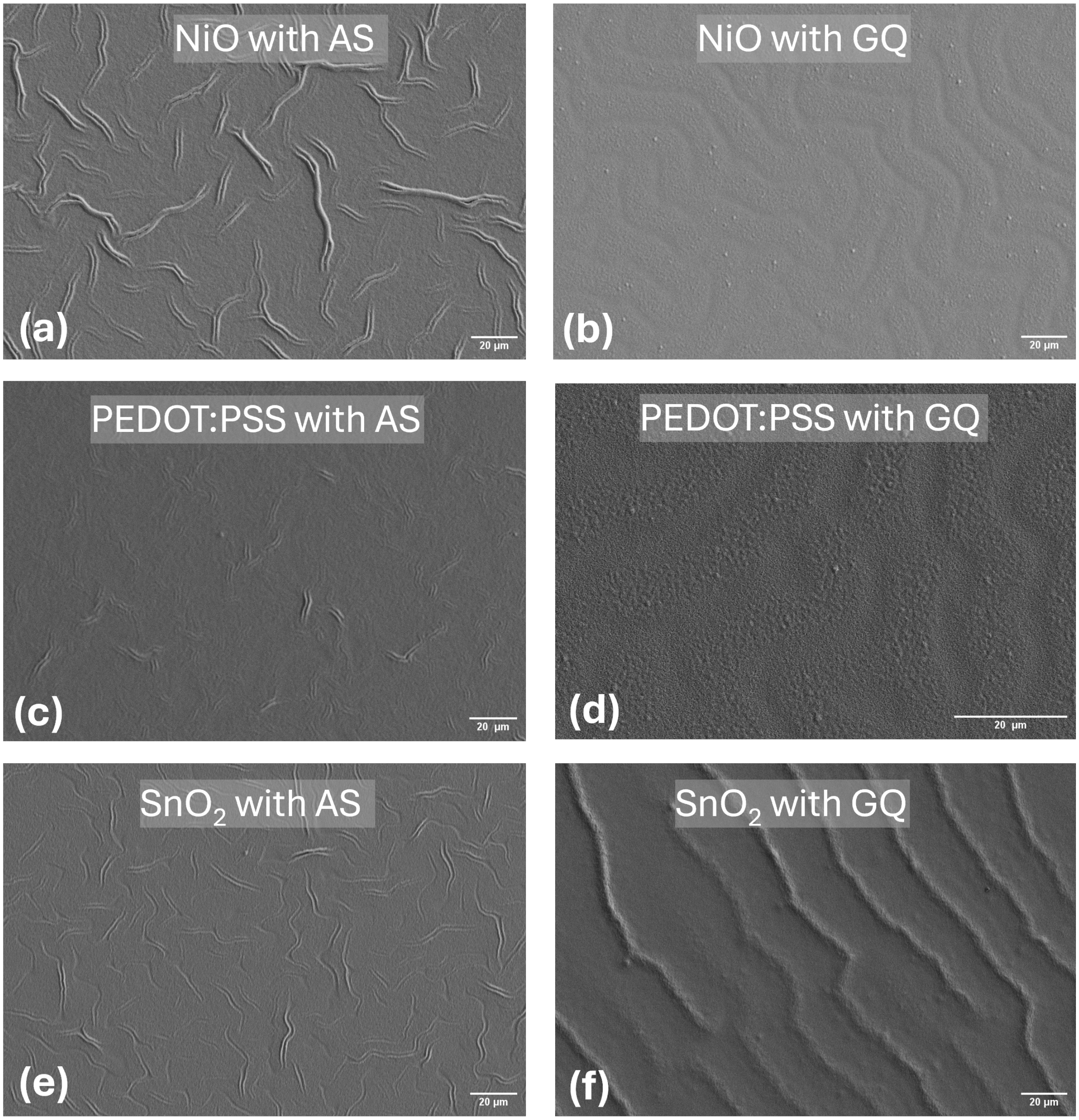

Figure S15: (a) SEM image of NiO as HTL with AS perovskite film. (b) SEM image of NiO as HTL with GQ perovskite film. (c) SEM image of PEDOT:PSS as HTL with AS perovskite film. (d) SEM image of PEDOT:PSS as HTL with GQ- perovskite film. (e) SEM image of SnO_2 as ETL with AS perovskite film. (f) SEM image of SnO_2 as ETL with GQ perovskite film.

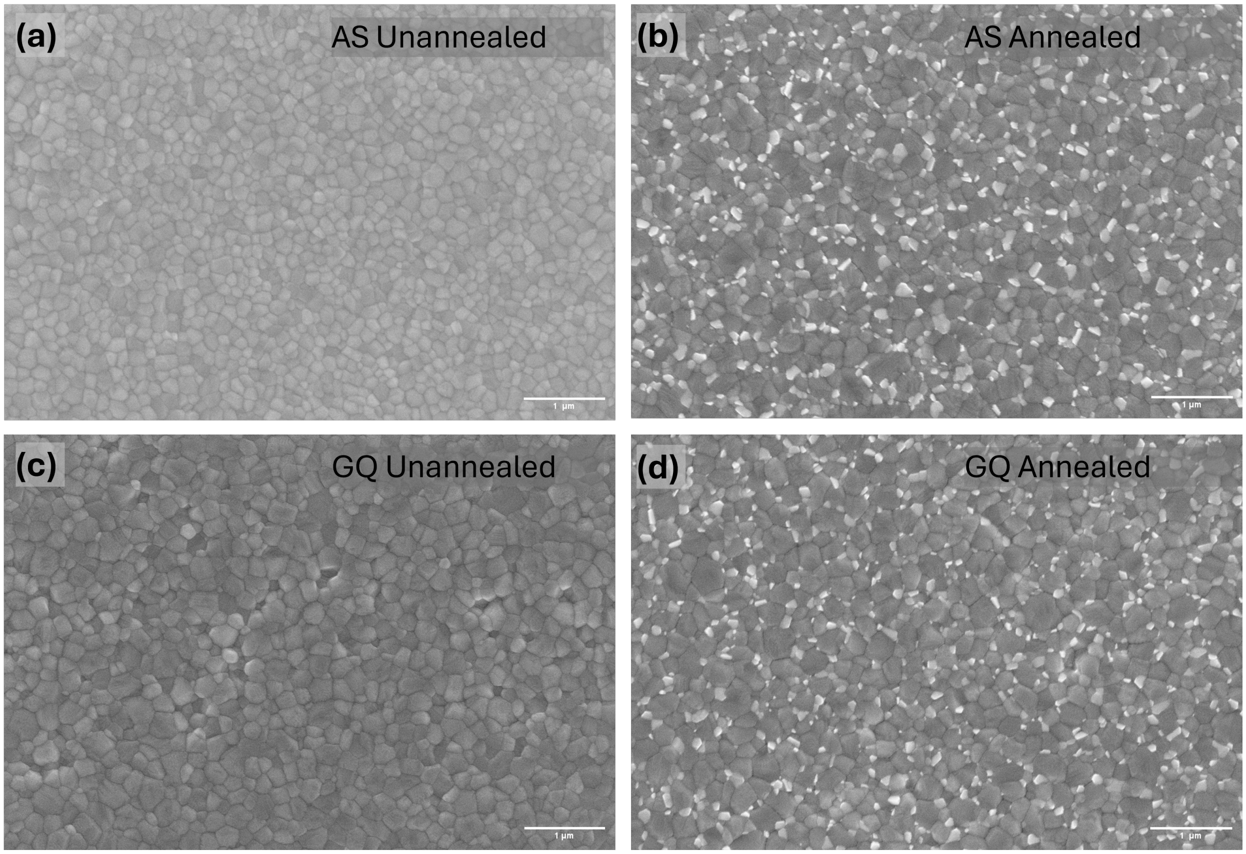

Figure S16: SEM images of half stack (ITO/HTL/perovskite) for (a) AS unannealed, (b) AS annealed, (c) GQ unannealed and (d) GQ annealed perovskite film.

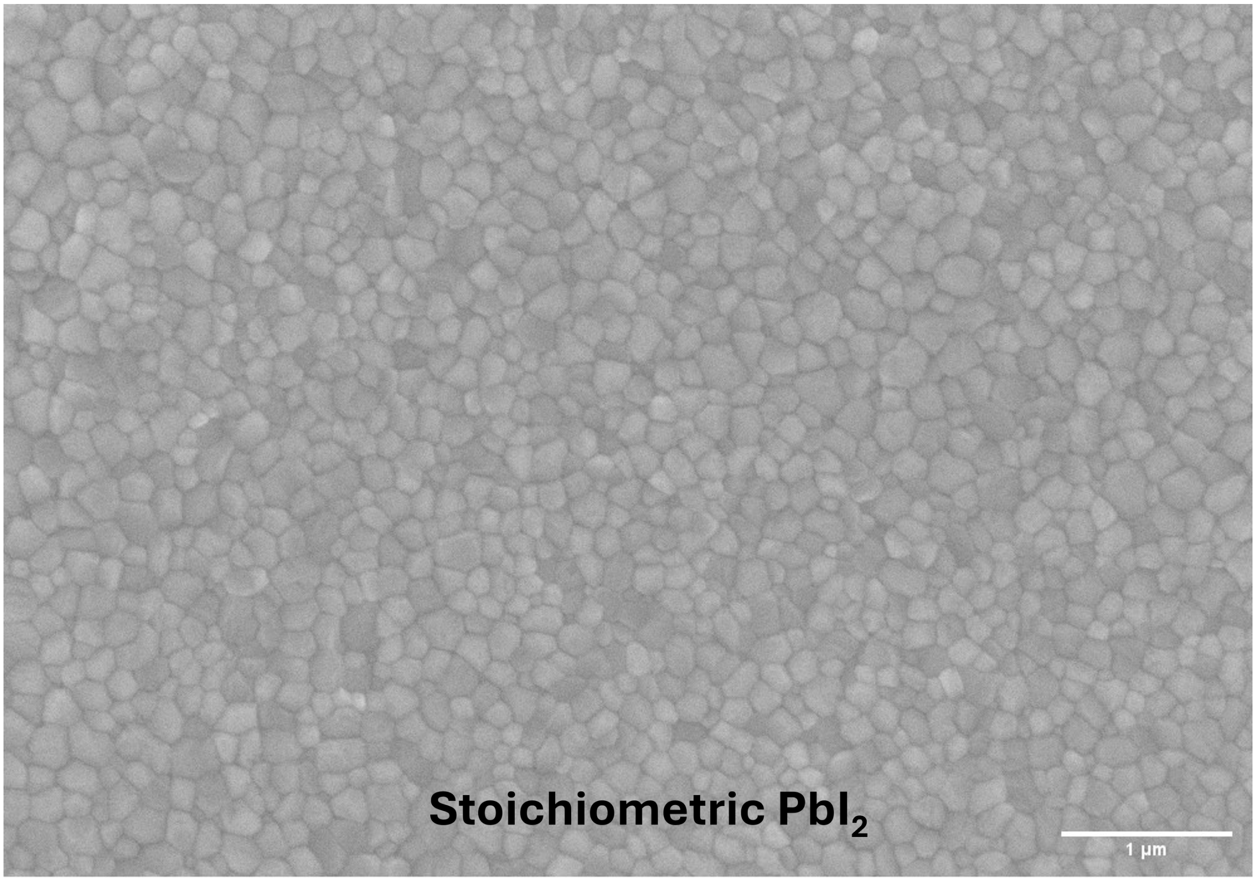

Figure S17: SEM image of GQ perovskite film with stoichiometric PbI_2 of a half stack (ITO/HTL/perovskite).

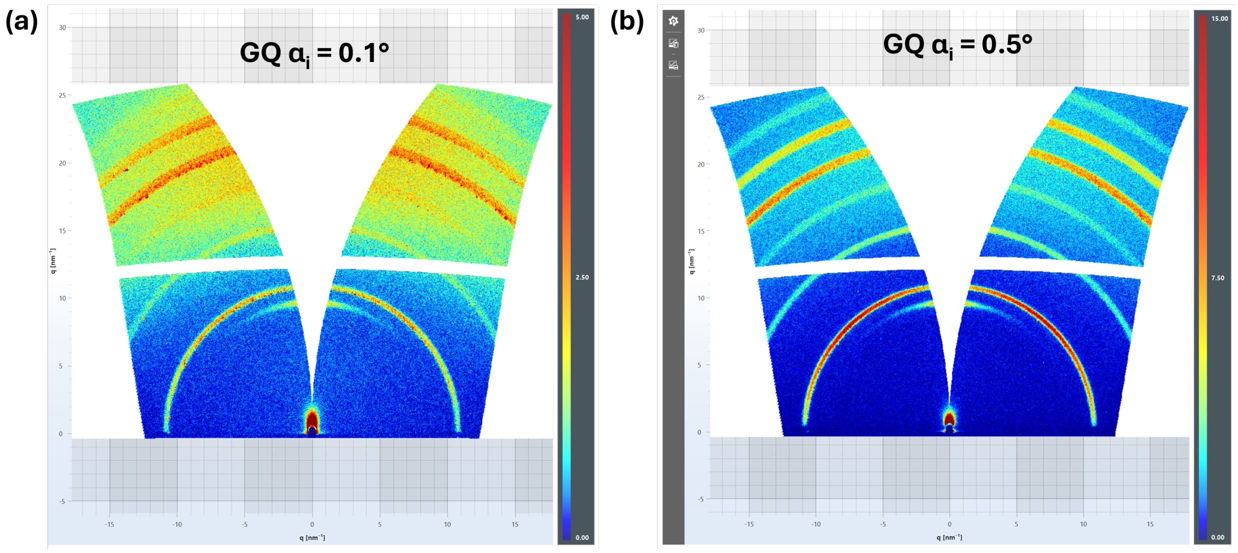

Figure S18: Transformed GIWAXS images for GQ samples with an incident angle of 0.1° (a) and 0.5° (b).

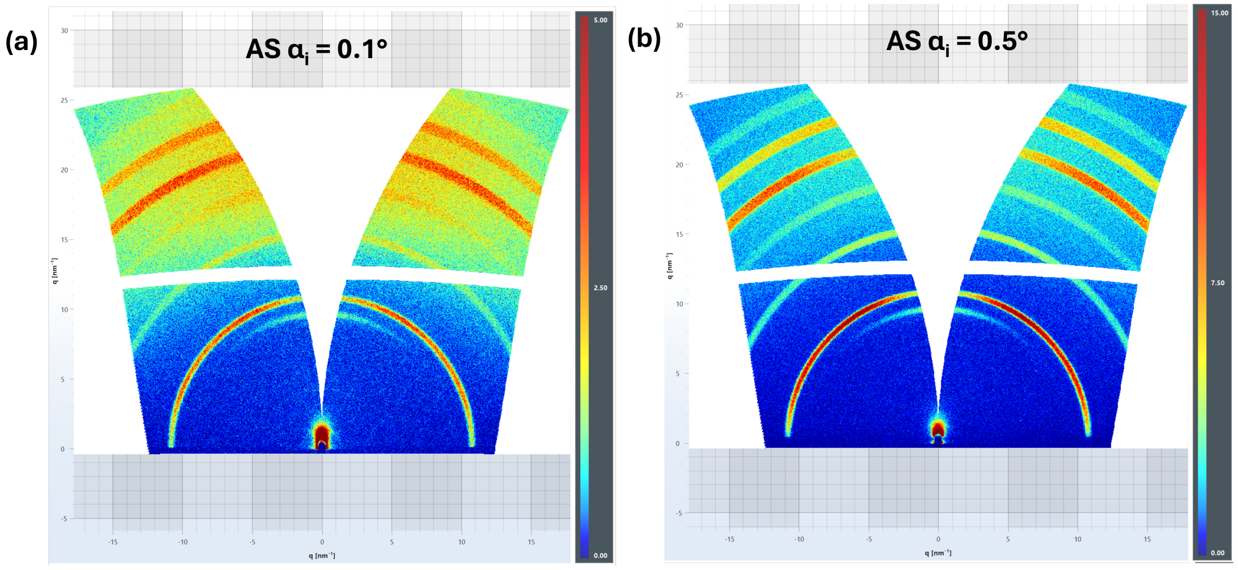

Figure S19: Transformed GIWAXS images for AS samples with an incident angle of 0.1° (a) and 0.5° (b).

XRD analysis

The experimental XRD diffractograms can be assigned to the pseudocubic Pm$\bar{3}$m symmetry, which displays only slight shifts from the P4mm symmetry. The comparison of the XRD

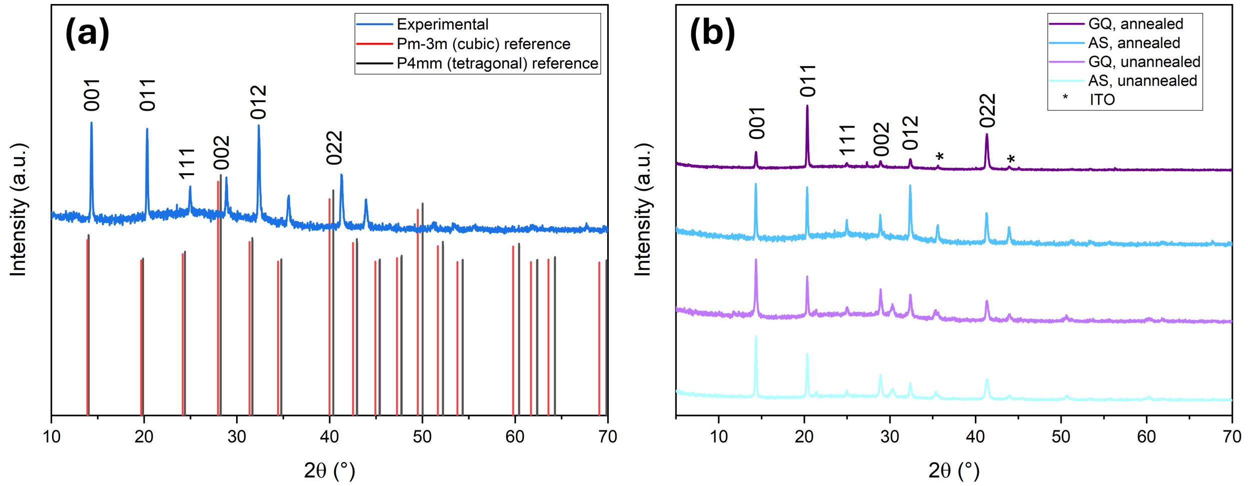

Figure S20: (a) XRD diffraction pattern of an exemplary $Cs_{0.15}FA_{0.85}Pb(I_{0.6}Br_{0.4})_3$ perovskite thin films compared to cubic and tetragonal perovskite reference patterns. The cubic and tetragonal perovskite reference patterns were generated by the CIF files retrieved from the Crystallography Open Database (COD) under the reference numbers 7236562 and 4335634, respectively. (b) Normalized XRD plots of unannealed and annealed AS and GQ perovskite films.

diffractograms of the four sample types shows minor changes in the preferential orientation of the crystals. These are more pronounced for the annealed GQ samples, which display a preferential orientation along the (011) plane rather than along the (001).

Further characterizations

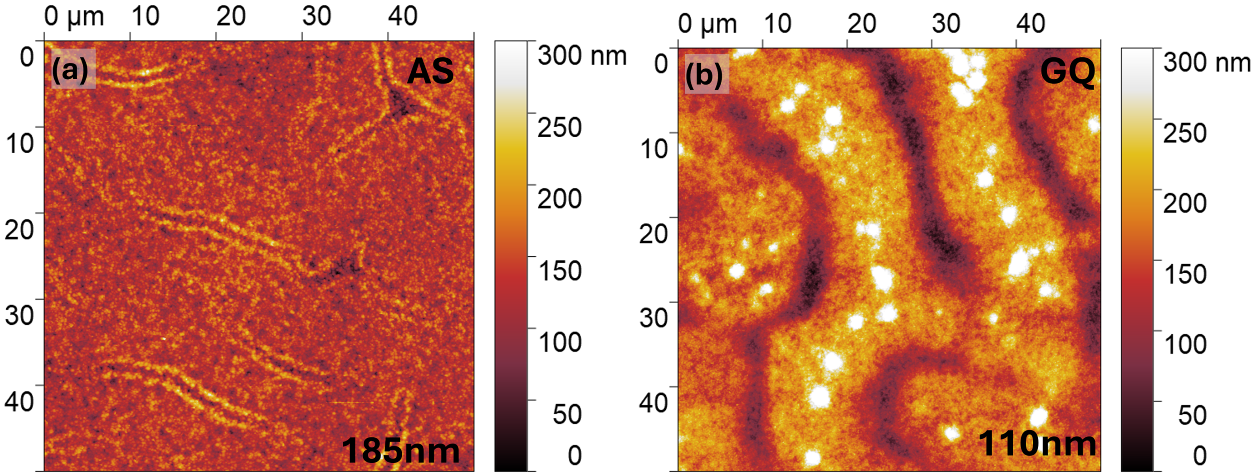

Figure S21: (a) Topography images of AS perovskite film and (b) GQ perovskite film.

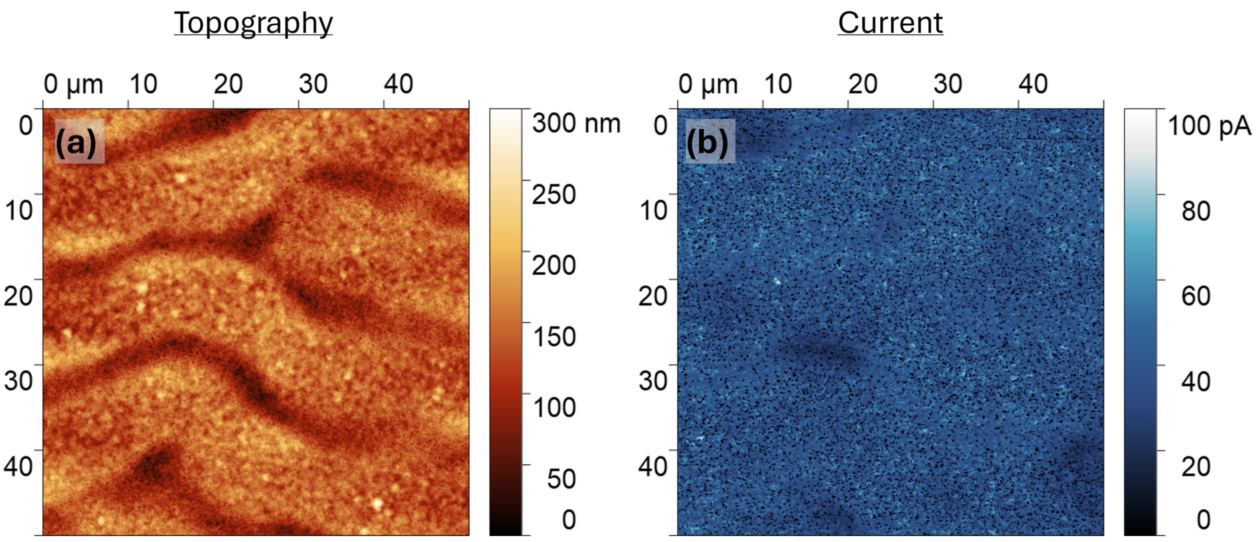

Figure S22: (a) Topography image of GQ perovskite film. (b) Current image of GQ perovskite film in dark mode.

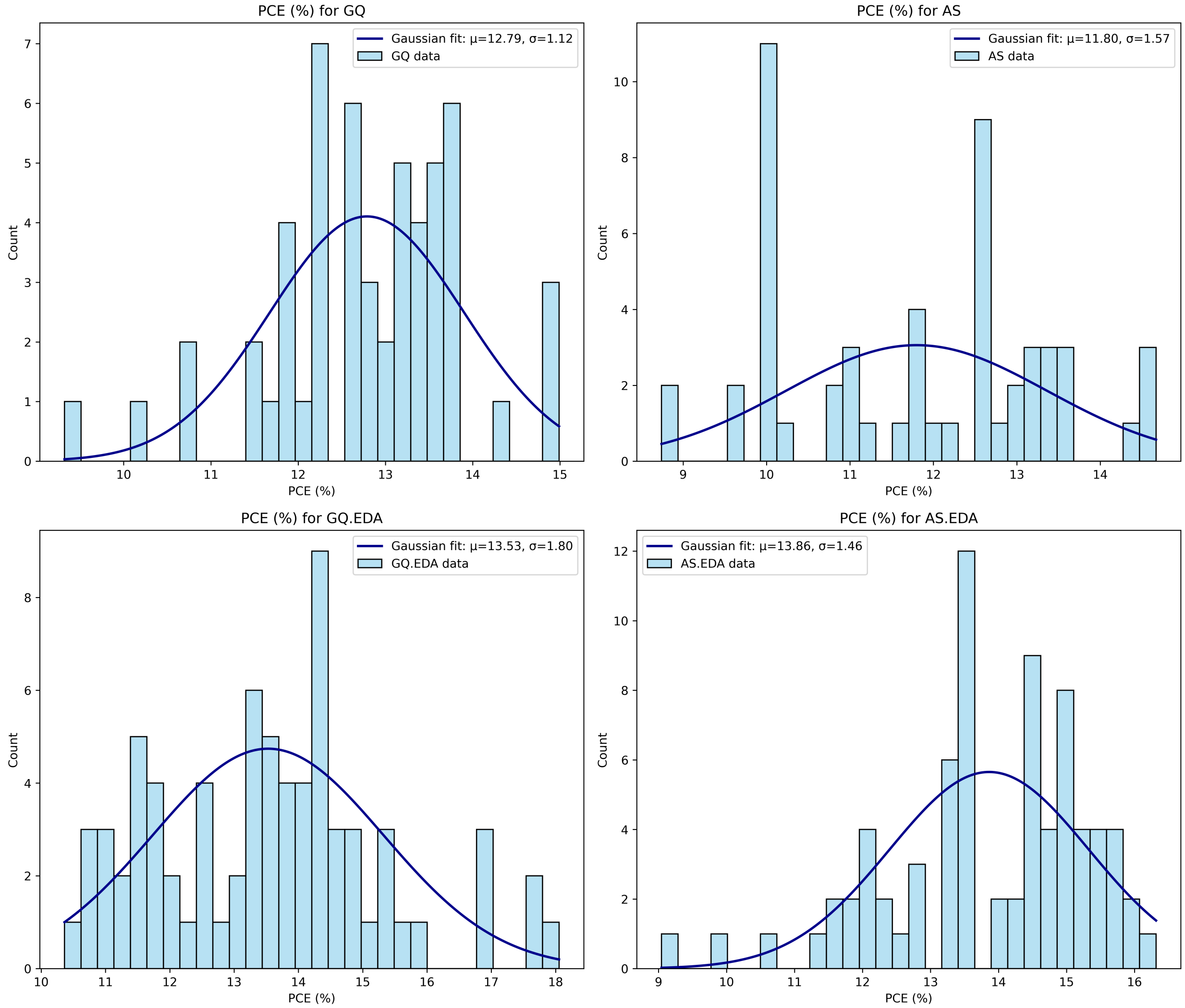

Figure S23: Statistical data showing PCE of four devices with mean and standard deviation values.

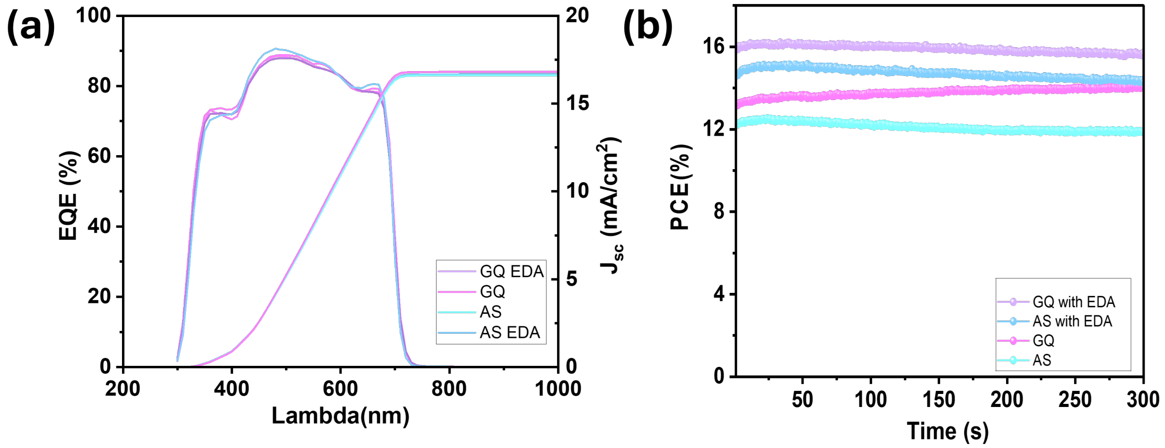

Figure S24: (a) EQE data with computed $J_{\mathrm{SC,EQE}}$ and (b) MPP tracking for the four different types of solar cell devices.

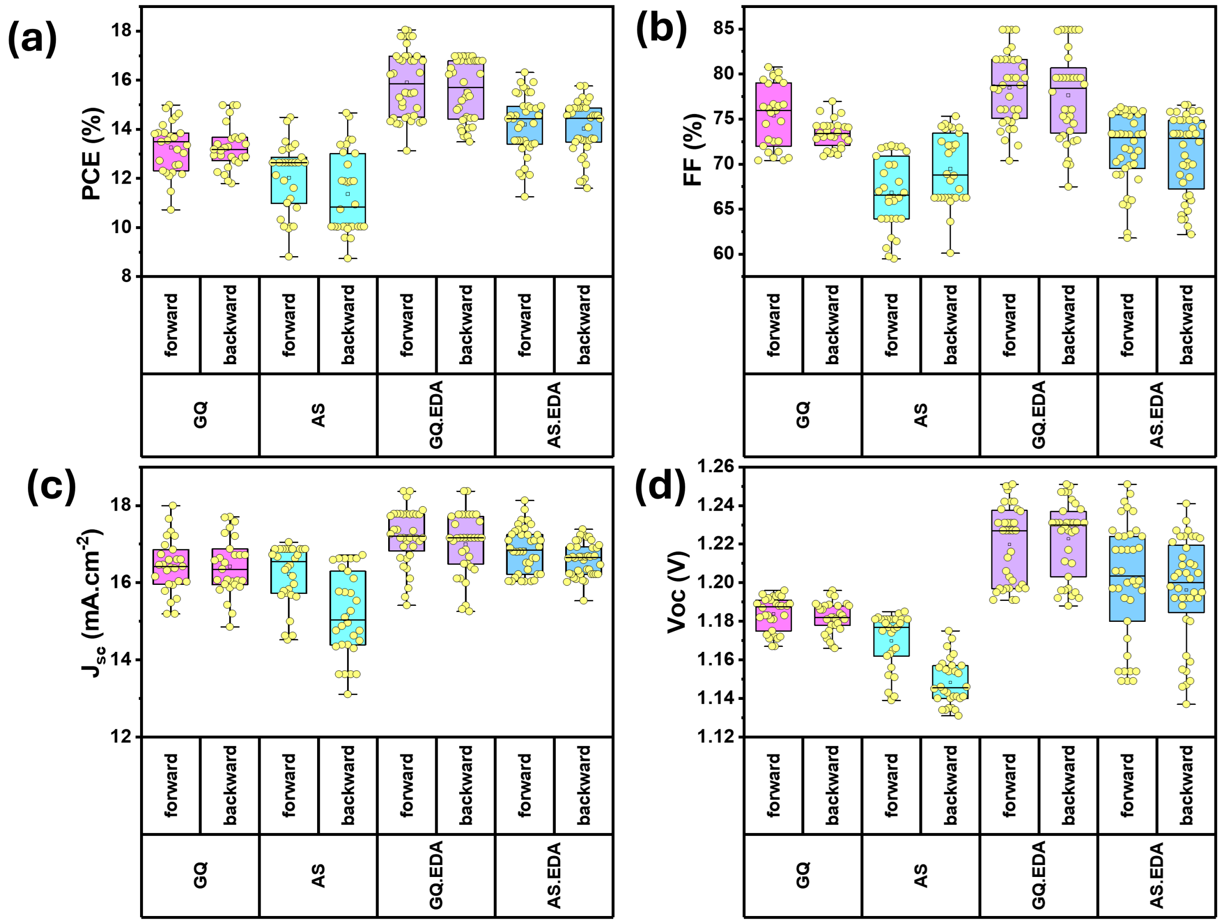

Figure S25: Boxplots of (a) PCE, (b) FF, (c) J_{SC} and (d) V_{OC} for four sets of solar cell devices.

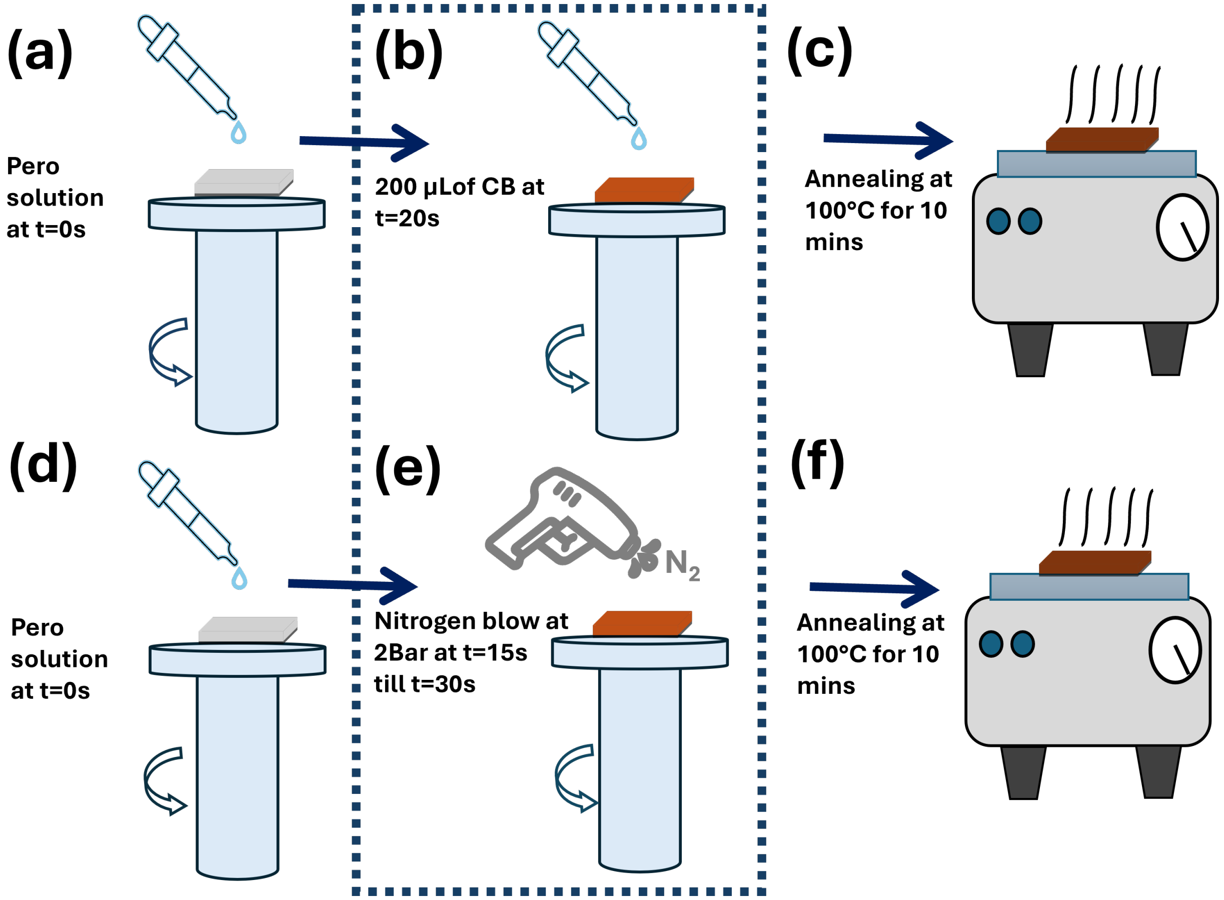

Figure S26: Schematic depiction of the two fabrication processes for perovskite thin films: (a-c) AS method and (d-f) GQ method

Table 1: PV parameters of champion cells for four types of tested groups

Device type	PCE (%)	V_{OC} (V)	J_{SC} (mA/cm^2)	FF (%)
AS	14.49	1.18	17.05	71.7
GQ	14.99	1.04	17.66	79.8
AS.EDA	15.92	1.20	17.51	76.0
GQ.EDA	17.88	1.23	17.78	81.6

In-situ transmission setup

The light source consists of a white-light laser (SuperK EXTREME EXU-6 from NKT Photonics A/S) combined with a filter box (SuperK SPLIT VIS from NKT Photonics A/S). Only the visible portion of the white light is utilized for the measurements. To adjust the

intensity, additional neutral density and short-pass filters are applied, ensuring that the maximum intensity at the sample position does not exceed 1 mW/cm^2. A custom-built spin coater, equipped with a hard drive motor capable of covering a wide range of rotational speeds, is used. A 3 mm hole is drilled through the motor axis, allowing the light to pass through and simultaneously defining the spot size on the sample. For spectral analysis, the AvaSpec-ULS2048CL-EVO spectrometer from Avantes BV is employed, which offers a recording speed of 380 μs per spectrum and a wavelength resolution of 1.4 nm. Additionally, the spectrometer provides precise timing information that is used for further data evaluation.

The motor and chuck assembly are housed in a chamber equipped with six gas inlets, enabling atmospheric modifications during spin coating. In this study, these inlets are used to purge the chamber with nitrogen for specific experiments. The entire setup, including data acquisition and synchronization, is controlled via an Arduino Mega.

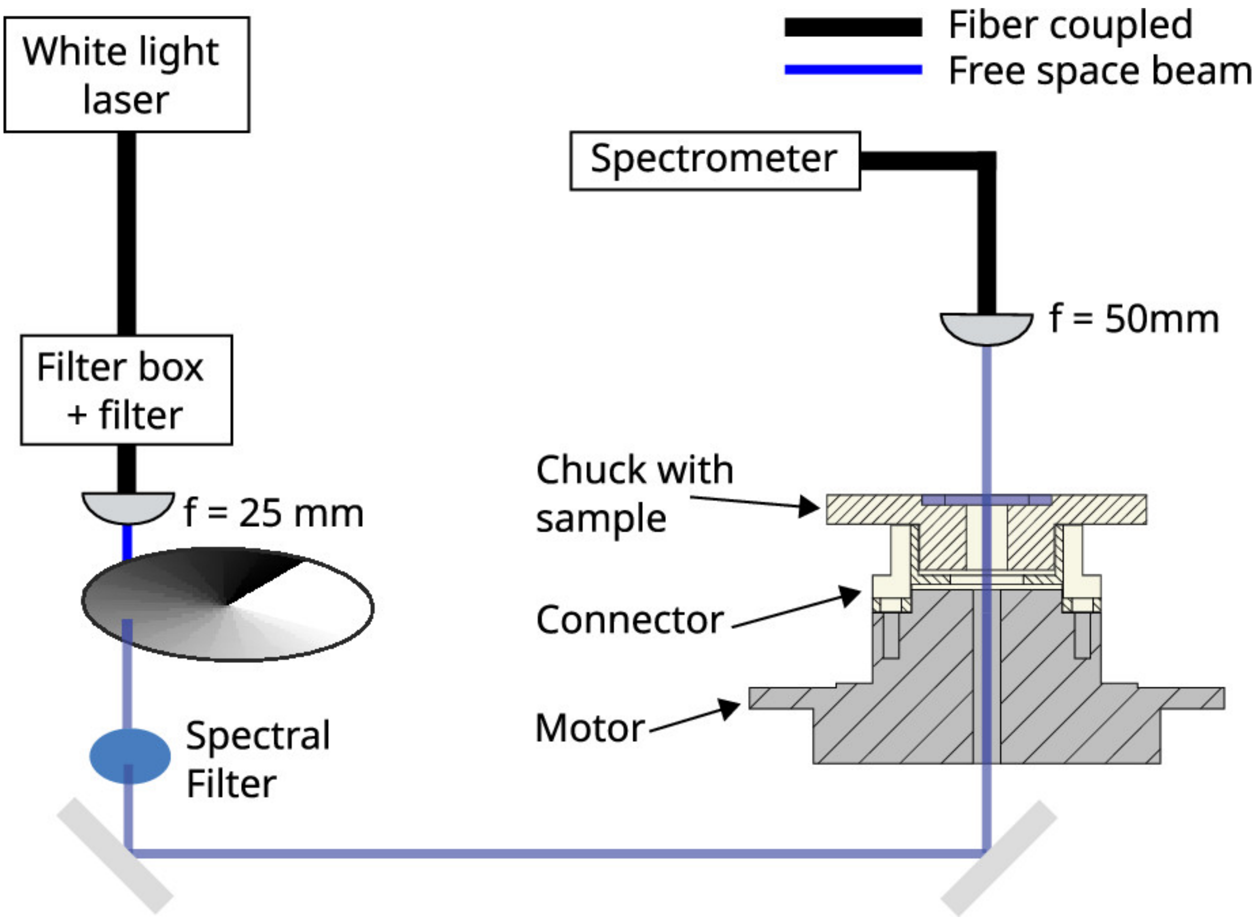

Figure S27: Schematic representation of the in situ spin coating transmission setup with permission from Raab et al.[3] Copyright (2022) American Chemical Society.

For each measurement, a dark spectrum and a reference spectrum of the sample are recorded to determine the transmittance during spin coating. The reference spectrum is taken while the spin coater is already running, but before the solution is applied, ensuring that sample movements during the startup phase do not affect the transmission data.

To minimize periodic noise caused by rotation, the transmission spectra are temporally smoothed using a moving average filter. The actual rotation speed is determined via fast Fourier transformation, allowing for precise filtering. Additionally, spectral smoothing along the wavelength axis is applied to enhance visualization. This setup enables the measurement of transmittance changes, which may differ from absorption changes due to reflection or scattering effects. This has two key implications: first, transmission data cannot be di-

rectly translated into absorption values. However, in most cases, the overall spectral shape provides a reasonable indication of the absorption behavior of the deposited layer. Second, transmittance values exceeding 100% may occur, indicating that the spin-coated layer acts as an anti-reflective coating. Such effects are observed in all transmission measurements and are corroborated by transfer matrix method simulations. Furthermore, the transmission results obtained with this setup align well with those from a commercial UV–vis spectrometer, demonstrating the suitability of this setup for in situ measurements.[3,4]

Grain size analysis

To access a grain size distribution, SEM images of AS- and GQ samples before- and after annealing were analyzed systematically. Five images per group, taken on different spots on the sample, were used for this analysis.

To identify grain boundaries, we employed holistically-nested edge detection (HED)[5,6] for shape recognition, followed by a series of post-processing steps. The HED algorithm was applied to the SEM images to enhance edge features, after which a thresholding operation was used to convert the output into a binary representation. The binary image was then skeletonized to refine the detected boundaries, and individual grain regions were labeled through connected component analysis. This automated approach allowed for accurate segmentation of grain boundaries while minimizing manual intervention.

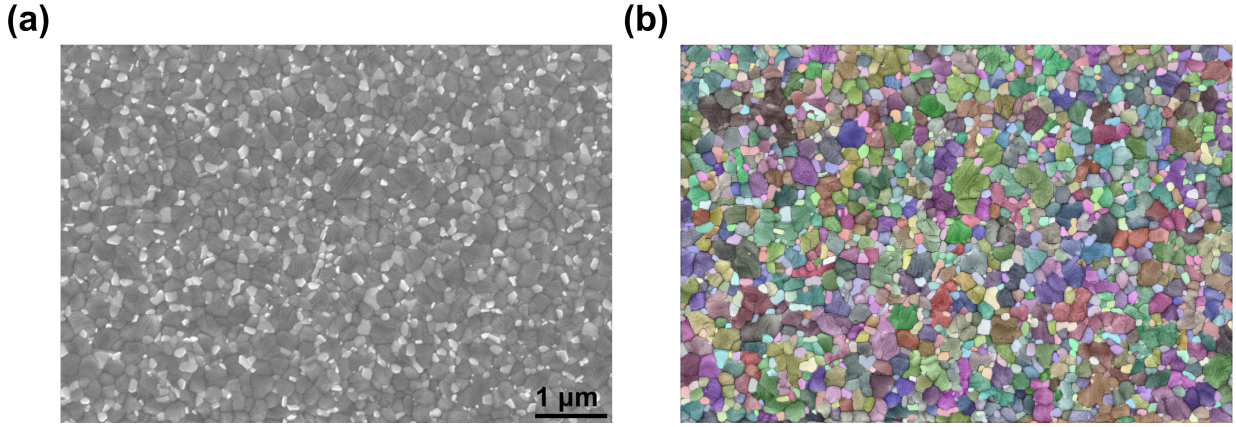

Figure S28: (a) Exemplary SEM as used for grain size extraction, (b) exemplary SEM image of the identified perovskite grains.

As shown in Figure S28 (a), the grains are reasonably well-resolved and can be identified well (Figure S28 (b)). As also visible in Figure S28 (a), a lighter variety of grains seems to be distinct from dark perovskite grains and is distributed all over the surface. This brighter species has been attributed to distinct PbI_2 domains. These brighter grains only appear *after* annealing, and tend to be much smaller than the average perovskite grain in the respective films. Simply extracting the grain sizes of *all* grains detected in these SEMs leads to a distorted distribution, as these smaller grains are (in part due to their size) more numerous than the darker perovskite grains and, thus, skew the histogram towards smaller grain sizes.

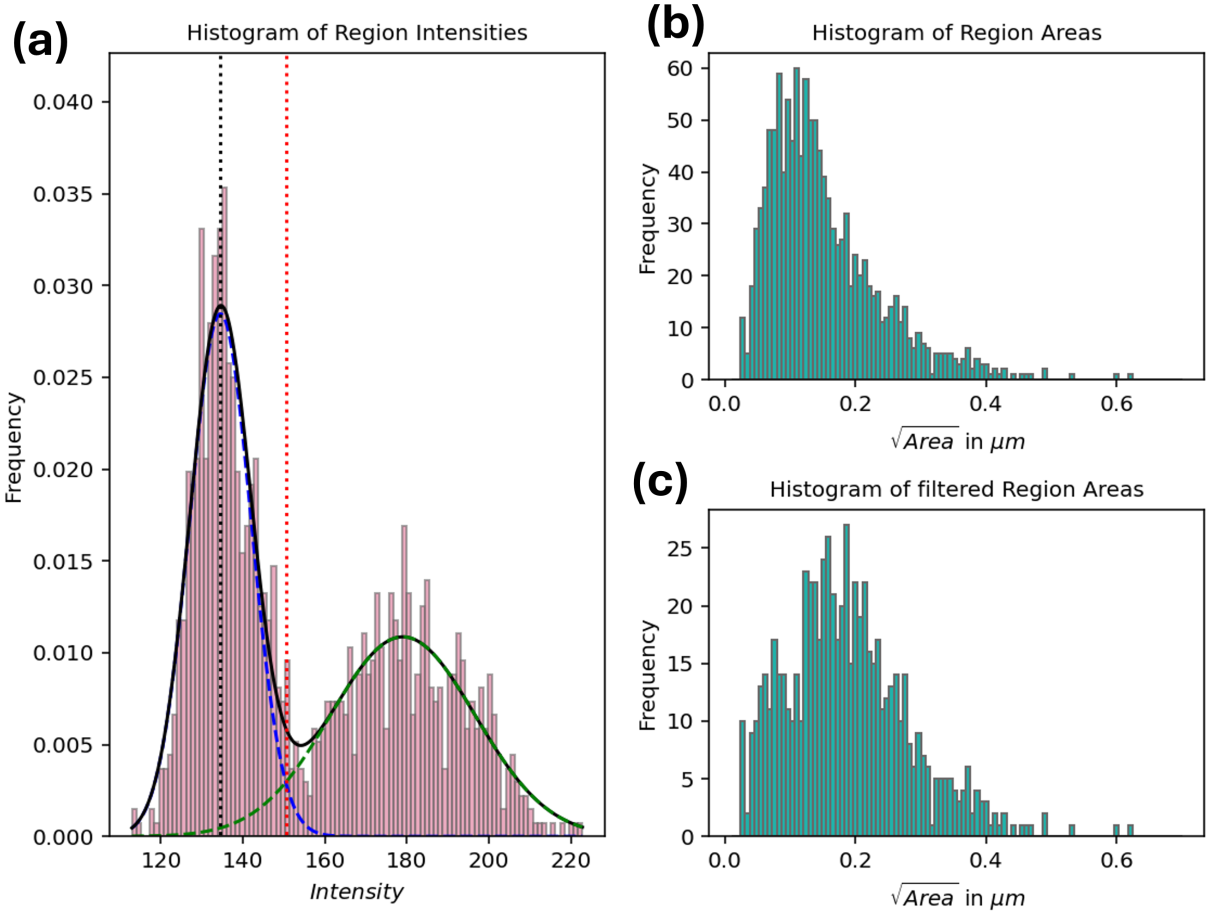

Figure S29: (a) Histogram of the average pixel intensity. After annealing, there are two distinct peaks visible: one light and one darker grain variety. Two gaussians are fitted to the data. Their intersection (red line) is used to distinguished the two pixels varieties. Only the darker species is used to calculate the average grain sizes. (b) Histogram of detected grain sizes before and (c) after removing of all grains of the lighter variety.

We chose to exclude the lighter variety from our analysis. This is justified, as we are interested in a comparison of the actual perovskite grain sizes, not of other residue phases present on the film.

In Figure S29 (a), the histogram of the average pixel intensities within all detected grains is shown. Two separate intensity peaks are clearly distinguishable, separating the two grain species from each other. To quantify this distinction, two gaussians are fitted into the histogram. The point of their intersection is used as a criterion to separate ’light’ grains from ‘dark’ grains. In Figure S29 (b) and (c), the grain size histograms of the same SEM

image is shown before (b) and after (c) excluding all grains lighter than the ‘cutoff’ intensity identified by the gaussian intersection. This is done for all images of films after annealing. As there are no lighter grains before annealing, this extra filtering step is omitted for those samples.

It should be noted that while both grain identification and the filtering of lighter grains work reasonably well, neither process is completely free of occasional artifacts. Such artifacts show up both as very large grains, where the boundary between two grains has not been identified properly, and as smaller ’grains’ that are actually not separate from other grains at all. The latter sometimes arise at and around grain boundaries, when the intensity gradients are somewhat unclear and tend to be very small. Especially for the annealed samples, where there is a multitude of light, small PbI_2 grains, such small artifacts are more frequent, as the grain boundaries in between the smaller grains often follow complicated shapes. Overall, though, the grains are identified well enough to reflect general trends in the grain sizes between the sample groups.

References

(1) Otsu, N. A Threshold Selection Method from Gray-Level Histograms. *IEEE Transactions on Systems, Man, and Cybernetics* **1979**, *9*, 62–66.

(2) Lee, T.; Kashyap, R.; Chu, C. Building Skeleton Models via 3-D Medial Surface Axis Thinning Algorithms. *CVGIP: Graphical Models and Image Processing* **1994**, *56*, 462–478.

(3) Raab, T.; Mayer, T.; Seewald, T.; Schmidt-Mende, L. Resolving the Spin Coating Process via In Situ Transmission Measurements. *The Journal of Physical Chemistry C* **2022**, *126*, 19542–19548.

(4) Raab, T. In-Situ Characterization of Film Formation in Solution-Processed Solar Cells.

http://nbn-resolving.de/urn:nbn:de:bsz:352-2-wbvaypmve3nn7, 2023; University of Konstanz.

(5) Xie, S.; Tu, Z. Holistically-nested edge detection. Proceedings of the IEEE international conference on computer vision. 2015; pp 1395–1403.

(6) Jia, Y. Caffe: An Open Source Convolutional Architecture for Fast Feature Embedding. http://caffe.berkeleyvision.org/, Accessed: April 2025.
